# Comparative genomic analysis of 5M^g^ chromosome of *Aegilops geniculata* and 5U^u^ chromosome of *Aegilops umbellulata* reveal genic diversity in the tertiary gene pool

**DOI:** 10.3389/fpls.2023.1144000

**Published:** 2023-07-13

**Authors:** Inderjit S. Yadav, Nidhi Rawat, Parveen Chhuneja, Satinder Kaur, Christobal Uauy, Gerard Lazo, Yong Q. Gu, Jaroslav Doležel, Vijay K. Tiwari

**Affiliations:** ^1^ Department of Plant Sciences and Landscape Architecture, University of Maryland, College Park, MD, United States; ^2^ School of Agricultural Biotechnology, Punjab Agricultural University, Ludhiana, India; ^3^ Crops Genetics, John Innes Centre, Norwich, United Kingdom; ^4^ Agricultural Research Service, United States Department of Agriculture (USDA), Albany, CA, United States; ^5^ Centre of Plant Structural and Functional Genomics, Institute of Experimental Botany, Olomouc, Czechia

**Keywords:** *Aegilops geniculata*, wheat, gene, homoeologous, resistance, disease, sequencing, *Aegilops umbellulata*

## Abstract

Wheat is one of the most important cereal crops for the global food security. Due to its narrow genetic base, modern bread wheat cultivars face challenges from increasing abiotic and biotic stresses. Since genetic improvement is the most sustainable approach, finding novel genes and alleles is critical for enhancing the genetic diversity of wheat. The tertiary gene pool of wheat is considered a gold mine for genetic diversity as novel genes and alleles can be identified and transferred to wheat cultivars. *Aegilops geniculata* and *Ae. umbellulata* are the key members of the tertiary gene pool of wheat and harbor important genes against abiotic and biotic stresses. Homoeologous-group five chromosomes (5U^u^ and 5M^g^) have been extensively studied from *Ae. geniculata* and *Ae. umbellulata* as they harbor several important genes including *Lr57, Lr76, Yr40, Yr70, Sr53* and chromosomal pairing loci. In the present study, using chromosome DNA sequencing and RNAseq datasets, we performed comparative analysis to study homoeologous gene evolution in 5M^g^, 5U^u,^ and group 5 wheat chromosomes. Our findings highlight the diversity of transcription factors and resistance genes, resulting from the differential expansion of the gene families. Both the chromosomes were found to be enriched with the “response to stimulus” category of genes providing resistance against biotic and abiotic stress. Phylogenetic study positioned the M genome closer to the D genome, with higher proximity to the A genome than the B genome. Over 4000 genes were impacted by SNPs on 5D, with 4-5% of those genes displaying non-disruptive variations that affect gene function.

## Introduction

The United Nations’ current projections for global human population growth are alarming, with 8.6 billion people by 2030 and 9.8 billion people by 2050 (https://www.un.org). Being a staple source of calories for around 40% of the human population, common wheat (hexaploid bread wheat; 2n=6x=42; *Triticum aestivum* L.) is a critical crop for global food security (http://www.fao.org/worldfoodsituation/csdb/en/). Wheat faces continuous threats from changing climatic conditions and evolving biotic and abiotic stresses (Enghiad et al., 2017). To meet the ever-increasing demands, there is a need to increase wheat production and breed for high-yielding and more resistant cultivars (The International Wheat Genome Sequencing Consortium et al., 2018). To meet these challenges, genetic improvement of crop plants is the most sustainable agricultural approach, requiring continuous identification, characterization, and deployment of agronomically important genes and their useful allelic variants.

The hexaploid bread wheat has a complex genome architecture, consisting of three sub-genomes (A, B, and D) arising from different hybridization events. The first hybridization resulted in tetraploid wheat *Triticum turgidum* (2n=4x=28, AABB), where the A genome was contributed by the diploid progenitor *Triticum urartu* (2*n* = 2*x* = 14; AA). *Aegilops speltoides* (2n=14, SS) is thought to be a potential donor for the B genome. A subsequent hybridization between *T. turgidum* and *Aegilops tauschii* (DD) leads to the emergence of hexaploid wheat *Triticum aestivum* (2*n* = 6*x* = 42, AABBDD) (The International Wheat Genome Sequencing Consortium et al., 2018). The wheat gene pool is divided into three major categories based on the genomic constitution of the species i.e., primary, secondary, and tertiary. The primary gene pool consists of closely related species with shared genomes, diploid and tetraploid progenitors. The secondary gene pool is made up of species sharing at least one of the genome with wheat and consists of polyploid *Triticum* and *Aegilops* species. Species carrying non-homologous genomes are part of the tertiary gene pool (Chaudhary et al., 2014). *Aegilops* species are compatible with wheat and can be effectively used in trait enhancement using chromosome engineering approaches (Fernandez-Calvin and Orellana, 1992; Zhang et al., 1996).

*Aegilops geniculata* (2n=4x=28, M^g^M^g^U^g^U^g^) and *Aegilops umbellulata* (U^u^U^u^) are members of the tertiary gene pool and show wide distribution in the Mediterranean Basin (Arrigo et al., 2010) and Western Asiatic region (Meimberg et al., 2009), respectively. Leaf rust resistance (*Lr57)*, stripe rust resistance (*Yr40*) (Kuraparthy et al., 2007), stem rust resistance (*Sr53)* (Liu et al., 2011), and powdery mildew resistance (*Pm29*) (Zeller et al., 2002) genes have been introgressed into bread wheat over the years from *Ae. geniculata.* Similarly, *the* rust resistance gene *Lr*9 (Sears, 1956)*, Lr76 and Yr70* genes have been introgressed from *Ae. umbellulata* (Bansal et al., 2017) and utilized commercially (Sharma et al., 2021). The U- and M-genomes of *Aegilops* species are also a rich source of genes for nutritional quality (Bálint et al., 2001). Introgression of 2U^g^, 4U^g^, 5U^g^, 7U^g^, 2M^g^ and 7M^g^ chromosomes of *Ae. geniculata* increased the seed protein content of bread wheat (Rakszegi et al., 2017). Chromosomes 1U and 1M, increase the polymeric glutenin and demonstrate a positive effect on wheat dough strength (Garg et al., 2016; Rakszegi et al., 2017).

The high-throughput and cost-effective next-generation sequencing technology holds the potential to generate a vast number of molecular markers for the detection of alien introgression (Akhunov et al., 2009; van Poecke et al., 2013; Winfield et al., 2016). In polyploid crops such as wheat, flow cytometric sorting of individual chromosomes reduces the genomic complexity of the whole genome (Doležel et al., 2012). Flow-sorting has been widely used for studying relationship of different chromosomes and wild-species introgressions i.e., *Ae. geniculata*, *Ae. umbellulata*, *Ae. comosa*, *Ae. biuncialis, Ae. markgrafii*, *Ae. triuncialis*, *Ae.cylindrica*, *T. urartu*, *Ae. speltoides*, *Ae. tauschii* (Molnár et al., 2011; Molnár et al., 2014; Tiwari et al., 2014; Bansal et al., 2020). With a speedy pace of delivery of genomic resources and availability of germplasm from distant tertiary gene pool members, it becomes increasingly important to analyze gene level variations between cultivated wheat germplasm and its distant relatives. Understanding these gene-based variations will allow us to employ new tools such as gene editing to perform the next generation of crop improvement.

In the present study, we used the DNA sequences from the flow-sorted 5M^g^ chromosome of *Ae. geniculata* and 5U^u^ chromosome of *Ae. umbellulata* available in the public database to study the gene evolution of homologous chromosomes in comparison to the wheat genome (Chinese Spring assembly and the available wheat cultivar genomes) (Tiwari et al., 2014; Tiwari et al., 2015; Bansal et al., 2020). These two chromosomes were selected for being the source of the rust resistance gene (*Lr57*, *Lr76*, *Yr40*, *Lr70*, and *Sr53*). The study aims to identify the pattern of gene evolution for resistance genes and transcription factors in 5M^g^ and 5U^u^ chromosomes with respect to the homoeologous genes in modern wheat cultivars.

## Materials and methods

We downloaded the flow-shorted 5M^g^ chromosome sequence for the accession TA7670 from the SRA database (SRX1167449). The Flow-sorted chromosome sequence of 5U**^u^** was provided by Dr. Parveen Chhuneja (personal communication). SRA toolkit was used to partition the SRA file of 5M^g^ into individual fastq files (The SRA Toolkit Development Team). Fastqc was employed to check the quality of the raw reads (Andrews, 2010). Trimmomatic was used to remove adaptor sequences and filter low-quality reads (Bolger et al., 2014). The *de novo* sequence assembly was performed using SOAPdenovo2 at four different k-mers of k41, k51, k61, and k71 (Luo et al., 2012). BBmap was used to generate assembly statistics (Bushnell, 2014). The quality of the assembly was assessed using BUSCO conserved genes from poales_odb10 (n:4896) (Simão et al., 2015). Contigs shorter than 500bp were removed from the assembly. We used the hierarchical repeat finding approach, where the initial repeat finding was performed with Poaceae-specific repeats from Repbase (Bao et al., 2015). Genome-specific *de-novo* repeat families were identified using RepeatModeller (Flynn et al., 2020). Poaceae-specific repeats and *de-novo* repeats were masked using RepeatMasker (Tarailo-Graovac and Chen, 2009). *Ab-initio* gene prediction was done using AUGUSTUS (Hoff and Stanke, 2019). Genes with a length more than or equal to 150 bp or 50 amino acids were selected for further study. Full-length genes were selected based on the presence of both transcription start and termination sites in the prediction results for the study.

The diamond BLAST was used to search for the homologs of predicted genes from the NCBI non-redundant database (Buchfink et al., 2021). Blast2GO was used for the assessment of gene ontology (GO) terms and function assignment (Conesa and Götz, 2008). A Hidden Markov Model (HMM) based tool, pfamscan, was used for protein domain identification using the PfamA database (Mistry et al., 2007). OrthoFinder was used to detect the orthologs (Emms and Kelly, 2019). UpsetR was used to visualize orthogroups (Conway et al., 2017). Mercator4 was used to assign MapMan functional annotations and pathways to *Ae. geniculata, Ae. umbellulata*, chr5A, chr5B, and chr5D (Schwacke et al., 2019). Transcription factors were annotated using PlantTFDB (Jin et al., 2017). The alignment program BWA was used to align 5M^g^ and 5U**^u^** reads to the Chinese Spring genome (Li and Durbin, 2010). The sam files were converted to bam format using samtools (Li et al., 2009). BCFtools was used for SNP calling and filtration (Danecek et al., 2021). SNPeff was used to determine the effect of mutations on the gene structure (Cingolani et al., 2012). Variant files were merged with the exome capture data from 890 wheat exomes (He et al., 2019). ggplot2 was used to generate a PCA plot from the merged vcf file for chr5D (Wickham, 2016). A syntenic map of 10Mb introgressed region was prepared using syntenyPlotteR (Farré et al., 2019). SSR markers were identified using MISA (Thiel et al., 2003). Primer3 was used to design primers for the SSR marker (Koressaar and Remm, 2007). Polymarker was used to design Kompetitive allele specific PCR (KASP) markers for 5M^g^ and 5U^u^ SNPs mapped on chromosome 5D of CS (Ramirez-Gonzalez et al., 2015).

### Comparison of gene structure between 5M^g^, 5U^u,^ and wheat

Two approaches were used to compare the gene structure between 5M^g^, 5U^u^ and 5D. In the first approach, single copy-orthologs were compared for the differences in gene structure based on the number of exons, number of introns, and gene length. In the second approach, full-length genes of 5M^g^ and 5U^u^ were mapped to the chromosome 5D of CS using exonerate program (Slater and Birney, 2005). The mapped fragments were filtered based on exonerate similarity score >1000. Genes mapping to a genomic region longer than 15kb were removed from the comparison.

### Phylogenetic analysis

For the phylogenetic study, a species tree was generated using the single copy-genes based on the conserved gene alignments of the species i.e., *Oryza sativa* spp. japonica, *Brachypodium distachyon*, *Hordeum vulgare*, *Secale cereale*, *T. urartu*, *T. turgidum*, *T. turgidum* ssp. *dicoccoides*, *Ae. tauschii*, D genome of hexaploid wheat species (*T. aestivum* cv. Chinese Spring, *T. aestivum* cv. Arinalrfor, *T. aestivum* cv. Jagger, *T. aestivum* cv. Julius, *T. aestivum* cv. Lancer, *T. aestivum* cv. Landmark, *T. aestivum* cv. Mace, *T. aestivum* cv. Mattis, *T. aestivum* cv. Norin61, *T. aestivum* cv. Stanley, *T. spelta).* Alignment free distance-based method implemented in JolyTree (Criscuolo, 2019) was used to generate phylogenetic tree from the chromosome level alignment of 5M^g^ and 5U**^u^** with the group 5D chromosome of different wheat cultivars from the 10+ wheat genome project (Walkowiak et al., 2020).

### Evolution of gene families

Ortholog assignment of the Orthofinder results was converted into a presence-absence matrix using the orthocount2bin program (Natsidis et al., 2021). Gene families were classified *via* all-vs-all BLASTp search of 5M^g^, 5U^u^, 5D (Chinese spring), and 5D chromosome of *Ae. tauschii* (Camacho et al., 2009). BLASTp results were clustered into gene families using MCL (Enright et al., 2002) and family size counts were determined. The species tree generated by Orthofinder was converted into an ultrametric tree. Computational Analysis of gene Family Evolution (De Bie et al., 2006) was used to identify gene families that have undergone significant expansion or contraction in the genome. Gene family evolution was studied for the defined classes of resistance genes and transcription factors.

### Differential expression analysis of NIL carrying 5M^g^ introgression

To compare the structure, expression, and sequence level variations between wheat and its homoeologous chromosomes (with respect to 5M^g^), we used RNAseq expression data of small translocations of chromosome 5M^g^ on wheat chromosomes 5D in leaf rust and stripe susceptible wheat cultivar WL711 from previous studies on leaf rust (Yadav et al., 2016) and a second RNAseq dataset was provided by Dr. Parveen Chhuneja (personal communication). The susceptible wheat cultivar WL711 and two introgression lines, pau16062 (syn. T756, WL711 + *Lr57/Yr40*) and pau16055 (syn. T598, WL711 + *Lr57/Yr40*), in the WL711 background, were inoculated with leaf rust. RNA samples were collected at 6-time intervals after inoculation: 0 HPI (control), 12, 24, 48, 72, and 96 HPI. Hisat2 was used to map filtered reads to the Chinese Spring (CS) genome (Kim et al., 2019). FeatureCounts was used for populating expression counts (Liao et al., 2014). Differential expression analysis was performed at respective HPI between the introgression lines (T756 and T598) and the susceptible cultivar WL711 using edgeR (McCarthy et al., 2012). WEGO was used to plot the gene ontology (Ye et al., 2018). Gene ontology enrichment was performed using goseq package (Young et al., 2010).

## Results

### Sequence assembly

**Figure 1:**
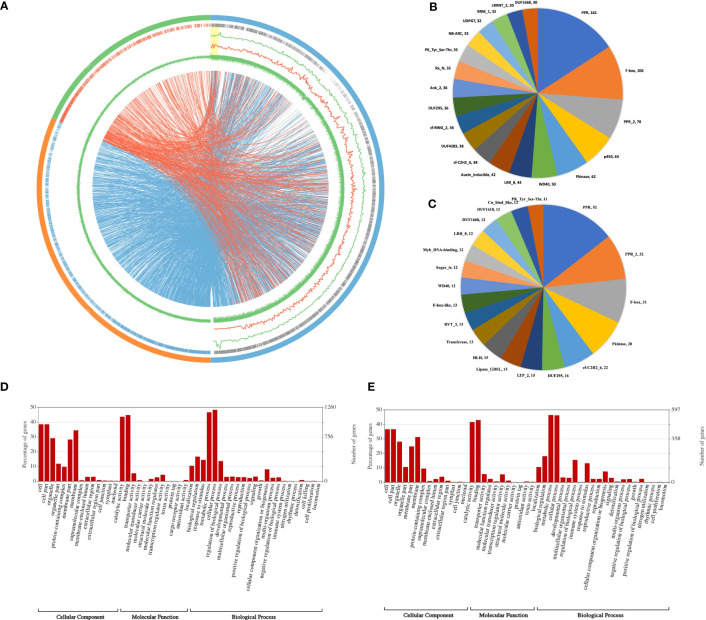
**(A)** Chromosome similarity plot of *Ae. geniculata* and *Ae. umbellulata* chromosomes in comparison to chromosome 5D of wheat. Outer circle: 5D (blue),5M^g^ (orange), 5U^u^ (green). Second circle shows the gene density. Line graphs 5M^g^ (green), and 5U^u^ (orange) show SNP density on 5D. GC content (inner green circle). Connecting lines display orthologous genes. **(B)** Top 20 protein family domains of *Ae. geniculata* (5M^g^) and **(C)** *Ae. umbellulata* (5U^u^). Classification of genes in gene ontology (GO) classes **(D)** *Ae. geniculata* (5M^g^) and **(E)** *Ae. umbellulata* (5U^u^).

For chromosome assembly in the present study, we utilized the 40 Gb (53X coverage) high-quality filtered data of *Ae. geniculata* (5M^g^ chromosome) downloaded from NCBI and 14.61 Gb (18X coverage) data of *Ae. umbellulata* (5U^u^ chromosome). The fragment size for 5M^g^ reads was 500 bp, while the fragment size for 5U^u^ reads was 600 bp. The highest N50 was obtained at k71 and k61 for 5M^g^ and 5U^u^, with an N50 of 1.078 kb and 559 bp, respectively. For chromosomes 5M^g^ and 5U^u^, a total of 675 Mb and 499 Mb of the sequence was assembled, respectively. We selected the contigs with a length of more than 500 bp, resulting in 397 Mb and 243 Mb of sequence for 5M^g^ and 5U^u^, respectively (**Table 1**). BUSCO-based assembly evaluation reported 736 and 383 complete BUSCO genes in 5M^g^ and 5U^u^, respectively (**Figure S1**). A total of 79 and 63 percent repetitive elements were detected using the hierarchical repeat identification approach; these included 77 and 60 percent repeats that were specific to *Poaceae* and 1.75 and 2.67 percent *de novo* repeats, in two assembled chromosomes 5M^g^ and 5U^u^, respectively (**Table S1**). Scaffolding using Ragoo (Alonge et al., 2019) resulted in 284 Mb of scaffolded contigs for 5M^g^, whereas for 5U^u^ only 63 Mb of the contigs were scaffolded (**Figure 1A**).

**Table 1:** Sequence assembly statistics of *Ae. geniculata* (5M^g^) and *Ae. umbellulata* (5U^u^).

Assembly	*Ae. geniculata* (5M^g^)				*Ae. umbellulata* (5U^u^)			
	Kmer 71		Gapfilling (>500bp)		Kmer 61		Gapfilling (>500bp)	
**Total no. of scaffold**	1,854,032		202,327		1,608,425		219,000	
**Total no. of contigs**	2,619,128		222,657		1,831,974		315,403	
**Genome in scaffold sequence**	675.070 Mb		397.503 Mb		499.800 Mb		243.154 Mb	
**Genome in contig sequence**	595.063 Mb (11.852% gap)		395.173 Mb (0.586% gap)		413.541 Mb (17.259% gap)		215.904 Mb (11.207% gap)	
**Main genome scaffold N50/L50:**	98246/1.078 Kb		27129/3.404 Kb		204532/559		57394/1.134 Kb	
**Max scaffold length:**	55.822 Kb		55.666 Kb		31.308 Kb		30.444 Kb	
**Max contig length:**	32.207 Kb		55.666 Kb		16.486 Kb		30.443 Kb	
**Contig length**	No of scaffold	No of bases	No of scaffold	No of bases	No of scaffold	No of bases	No of scaffold	No of bases
**All**	1,854,032	675,070,048	20,232,700	397,502,599	1,608,425	499,799,616	21,900,000	243,153,551
**1 Kb**	105,913	345,500,735	102,022	328,272,111	87,787	165,760,371	76,064	141,598,981
**25 Kb**	299	9,072,840	279	8,438,200	7	191,104	5	137,978
**50 Kb**	5	271,480	5	267,609	–	–	–	–
Repetitive elements								
			**Repetitive base**	**percentage**			**Repetitive base**	**percentage**
**Poaceae specific repeats**			308,149,023	77.52%			148,107,104	60.91%
**Novel *de novo* repeat**			6,947,009	1.75%			6,488,548	2.67%
**Total masked based**			315,096,032	79.20%			154,595,652	63.58%

### Gene prediction and annotation

Gene prediction tool AUGUSTUS was used to perform *de novo* gene prediction in scaffolds larger than 1 Kb. In total, 9,702 and 7,258 genes were predicted in 5M^g^ and 5U^u^, respectively (**Table 2**). Homologs were detected for 82% of genes in the two assemblies. Gene ontology (GO) terms were identified for 5,504 (57%) and 4,199 (58%) genes; KEGG enzymes were annotated for 2,389 (24%) and 1,952 (27%) genes, and protein domains were detected for 4,836 (50%) and 3,431 (48%) genes in 5M^g^ and 5U^u^, respectively. Because the total number of genes in the assemblies varied greatly, we focused our study on 5,100 and 2,592 full-length genes in 5M^g^ and 5U^u^, respectively.

**Table 2:** Genes predicted in *Ae. geniculata* (5M^g^) and *Ae. umbellulata* (5U^u^) using AUGUSTUS.

Prediction/Annotation	*Ae. genicualta* (5M^g^)		*Ae. umbellulata* (5U^u^)	
**Total no of predicted genes**	9,702		7,258	
**Full length genes with transcription start site and termination site**	5,100		2,592	
Functional annotation				
	No. of gene	Full length gene	No. of gene	Full length gene
**Blast homologs**	8,123	4,294	5,882	1,399
**Gene Ontology**	5,504	2,918	4,199	1,399
**Enzyme**	2,389	2,521	1,952	1,195
**Pfam**	4,836	4,302	3,431	1,730

A total of 4,294 (84%) and 1,399 (54%) full-length genes were found to have BLAST homologs in 5M^g^ and 5U^u^, respectively. These genes also included the full-length BUSCO genes. Protein family domains were identified for 4,302 (84%) and 1,730 (66%) genes in two chromosomes (**Figures 1B, C**). The major protein classes consisted of PPR, F-box, and Protein kinase. Gene ontology classes were assigned to 2,918 (57%) and 1,399 (54%) of the genes in 5M^g^ and 5U^u^, respectively (**Figures 1D, E**). In both chromosomes, the major portion of the genes were found to be involved in cellular activity, metabolic processes, binding, and catalytic activity. Additionally, it was discovered that both chromosomes were enriched for the “response to stimulus” category of genes. Nearly 2,521 (49%) and 1,195 (46%) of the genes in 5M^g^ and 5U^u^, respectively, were assigned to KEGG enzymes (**Table S2**).

### Transcription factor and Resistance gene

**Figure 2:**
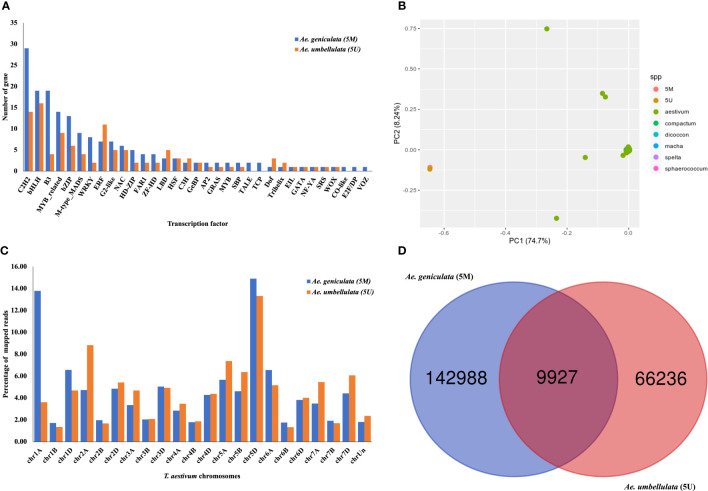
**(A)** Distribution of transcription factor classes identified from PlantTFDB. **(B)** Principal component analysis (PCA) of *Ae. geniculata* (5M^g^) and *Ae. umbellulata* (5U^u^) with tetraploid and hexaploid wheat accession. **(C)** Percentage alignment statistics of 5M^g^ and 5U^u^ reads on the complete genome of Chinese spring displaying partial read mapping on chromosomes other than 5D. **(D)** Shared high-quality homozygous SNPs of *Ae. geniculata* (5M^g^) and *Ae. umbellulata* (5U^u^).

In the 5M^g^ and 5U^u^ chromosomes, 176 and 109 transcription factors (TF) belonging to 33 and 28 TF classes were identified. There were approximately 38% more TF classes on chromosome 5M^g^ compared to chromosome 5U^u^ (**Figure 2A**; **Table S6**). The majority of TFs belong to C2H2, bHLH, B3, MYB-related, bZIP, M-type MADS, WRKY, and ERF classes and constitute 67% and 60% of the TF classes in 5M^g^ and 5U^u^, respectively. Protein family annotations based on NB-ARC and LRR domains revealed 78 (out of total 136) and 21 (out of total 80) full-length R-genes in 5M^g^ and 5U^u^, respectively.

### Relationship of 5M^g^ and 5U^u^ chromosomes with homoeologous bread wheat chromosomes

**Figure 3:**
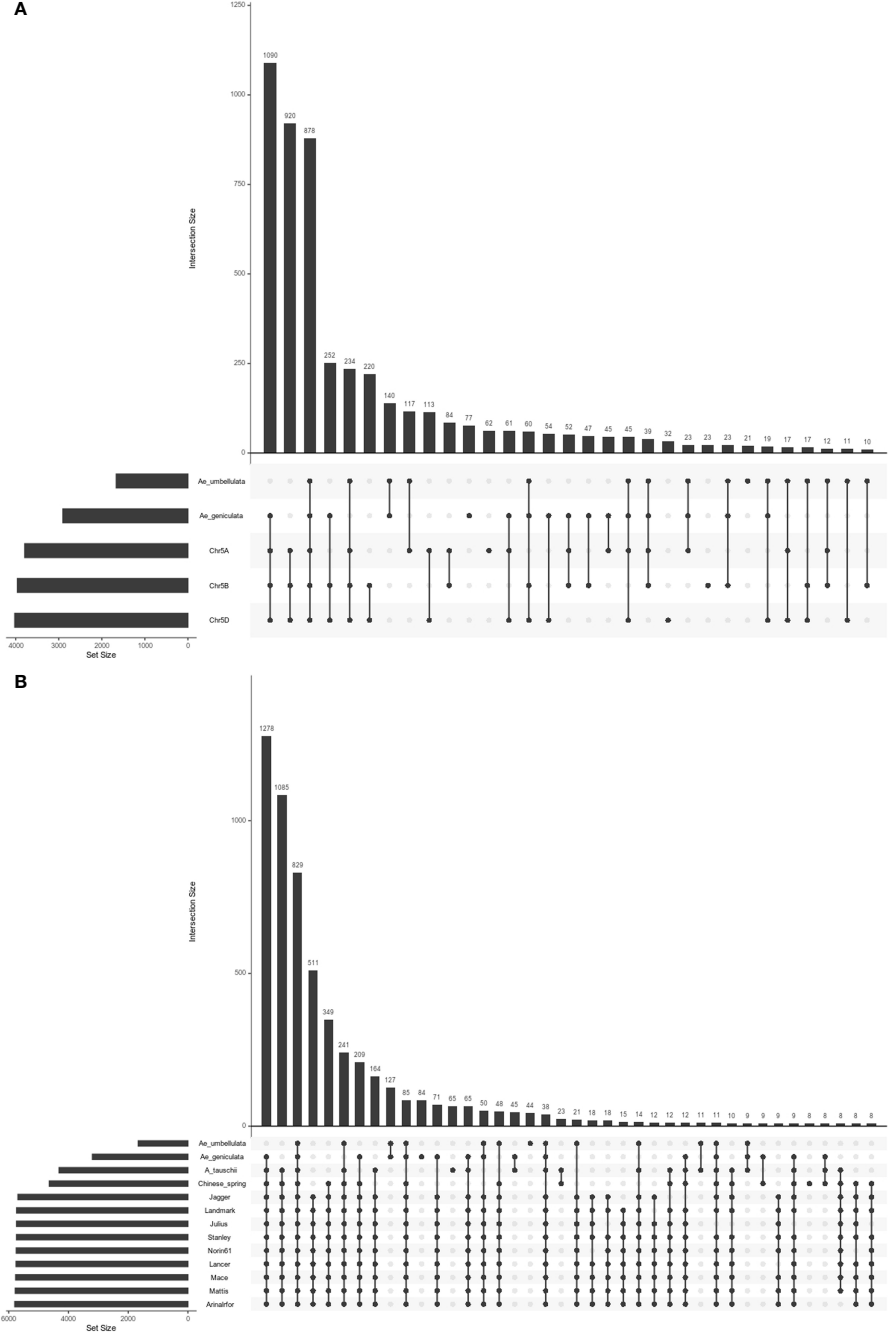
Upset plot of orthologous groups **(A)** orthogroups between *Ae. geniculata* (5M^g^), *Ae. umbellulata* (5U^u^), chr5A and chr5B and chr5D of Chinese spring. **(B)** orthogroups between *Ae. geniculata* (5M^g^), *Ae. umbellulata* (5U^u^), *Aegilops tauschii*, *T. aestivum* cv. Chinese Spring, *T. aestivum* cv. Arinalrfor, *T. aestivum* cv. Jagger, *T. aestivum* cv. Julius, *T. aestivum* cv. Lancer, *T. aestivum* cv. Landmark, *T. aestivum* cv. Mace, *T. aestivum* cv. Mattis, *T. aestivum* cv. Norin61, *T. aestivum* cv. Stanley.

Biologically related organisms share orthologous genes with similar functions. Chromosome 5M^g^ shared 1,400, 2,844, 3,223, and 3,266 homoeologous genes with the chromosomes 5U^u^, 5A, 5B, and 5D, while the chromosome 5U^u^ shared 1618, 1740, 1614, and 1604 orthologs with the chromosomes 5M^g^, 5A, 5B, and 5D. Number of shared orthogroups between the five chromosomes (5M^g^, 5U^u^, 5A, 5B, and 5D) are shown in **Figure 3A**. There were 878 shared orthogroups between 5M^g^, 5U^u^, 5A, 5B, and 5D chromosomes, where 77 and 21 orthogroups were specific to 5M^g^ and 5U^u^ chromosomes, respectively. A total of 140 orthogroups were common between the 5M^g^ and 5U^u^ genomes. While *Ae. umbellulata* shared 234 orthogroups with group 5 chromosomes of wheat, *Ae. geniculata* shared the highest number of orthogroups (1,090) with A, B, and D genomes. There were 514 single-copy orthologs between the five chromosomes. Singleton representation constituted 893 and 573 genes in *Ae. geniculata* and *Ae. umbellulata.* The orthologous relationship of 5M^g^ and 5U^u^ chromosomes with *Ae. tauschii* and ten other wheat cultivars is shown in **Figure 3B** (Luo et al., 2017; Walkowiak et al., 2020).

### Comparison of 5M^g^ and 5U^u^ chromosome sequences with wheat chromosome 5D

To see the divergence of the tertiary gene pool from the available germplasm resource, we compared the 5M^g^ and 5U^u^ with the exome capture data from a previous study (He et al., 2019). In the comparison with group 5D chromosomes from 890 diverse hexaploid and tetraploid wheat accessions, *Ae. geniculata* and *Ae. umbellulata* clustered into a separate group as depicted in **Figure 2B**. Based on the results of read mapping onto the whole wheat genome, the highest percentage of 5M^g^ reads were located on chr5D and chr1A, whereas the highest percentage of 5U^u^ reads mapped to chr5D and chr2A. Significantly lower read mapping was observed on the B genome (**Figure 2C**). According to phylogenetic studies and whole-genome alignment of reads, 5M^g^ and 5U^u^ chromosomes showed the most resemblance with group D, lesser with group A, and the least similarity to the B genome. **Figure S2** displays the coverage of uniquely mapped reads of *Ae. geniculata* and *Ae. umbellulata* on chromosome 5D of Chinese Spring. Both the chromosomes showed higher coverage of mapped reads in a 1Mb window. A dip in the coverage was observed at 150 Mb on chromosome 5D for the two aligned chromosomes. The distal region on the long arm of chromosome 5D displayed differential coverage for *Ae. geniculata* and *Ae. umbellulata*. The elevated region in the coverage plot indicates ancient introgression of the 5M^g^ fragment on chromosome 5D of the Chinese Spring, supported by the lower coverage for the corresponding segment from 5U^u^ of *Ae. umbellulata.*

From the read mapping, 7,776,990 (2.57%) of the concordantly paired reads of 5M^g^ and 3,625,329 (3.47%) reads of 5U^u^ mapped on the coding region of Chinese Spring (170Mb,1.17% of genome). Chr5D showed the highest coverage of reads mapped on the coding region, followed by chr5A and chr5B. Chr7D and Unassigned chromosome sequence exhibited good coverage of the 5U^u^ read, while Chr1A showed higher coverage of the 5M^g^ read (**Figure S3**). These results indicate genetic similarity and hybridization. A higher percentage of read mapping was observed in the intergenic region compared to the genic region on chromosome 5D. Read mapping to the coding region accounts for 2.45% at 5M^g^ and 3.08% at 5U^u^.

There were 249,041 and 114,485 high-quality SNPs identified for 5M^g^ and 5U^u^, respectively, with indels accounting for a larger portion (24%) of the detected variations. In 5M^g^, 25% of SNPs were heterozygous compared to only 15% in 5U^u^ (**Table 3**). **Figure 2D** displays the shared and unique SNPs between the two chromosomes mapped to chromosome 5D of Chinese Spring. Out of the total 5,545 high-confidence genes present on chromosome 5D, 5,090 and 4,482 genes were affected by the SNPs from the 5M^g^ and 5U^u^ reads, respectively. The majority of constituent variants were missense variants, accounting for 35% of all variant sites. An estimated 4 to 5% of the proteins were affected by the “moderate effect”, which leads to the non-disruptive variant that could alter protein efficacy. These non-disruptive variants may play an important role and may result in lowering the gene efficiency or may make a gene less effective to pathogens in wheat. A moderate impact on gene function was observed for 3,152 and 2,329 genes of 5D, impacted by 5M^g^ and 5U^u^ reads, respectively. There were 49 and 46 resistance genes impacted by 5M^g^ and 5U^u^ SNPs, respectively.

**Table 3:** Single-nucleotide polymorphism (SNPs) based on the *Ae. umbellulata* (5M^g^) and *Ae. geniculata* (5U**^u^**) chromosome-specific reads mapping to chromosome 5D of Chinese spring.

	*Ae. geniculata* (5M^g^)		*Ae. umbellulata* (5U^u^)	
	Number of identifed SNP	Number of homozygous alternate allele	Number of identifed SNP	Number of homozygous alternate allele
**Total number of variant sites**	249,041	152,915	114,485	76,163
**SNPs**	200,654	152,915	91,715	76,163
**Indels**	48,387	0	22,770	0
**SNP Transitions/Transversions**	1.74 (229667/131822)	1.75 (194622/111208)	1.72 (108316/63054)	1.72 (96338/55988)

### Differences in the gene structure between wheat and tertiary gene pool

Gene structure statistics of 5M^g^, 5U**^u^**, 5A, 5B, and 5D chromosomes are shown in **Table 4**. The longest genes in 5M^g^ and 5U**^u^** were 22 kb long, whereas those in Chinese Spring vary from 47 kb to 63 kb. The longest intron in 5M^g^ was 7.3 kb, in 5U^u^ it was 5.2 kb, and in Chinese Spring intron length ranged from 34 to 44 kb. Lesser number of exons and introns were observed per gene in 5M^g^ and 5U^u^ compared to that of Chinese Spring. Based on the fewer intron per mRNA and smaller mean intron length, we concluded a shorter gene length in alien germplasm compared to that of Chinese spring. We further explored the homoeologous gene structure between the chromosomes. A total of 712 orthologs with the same number of exons were found among 1,466 single-copy orthologs between 5M^g^ and 5D. We noted 61 out of 712 genes showed identical protein lengths in both 5M^g^ and 5D. Thirteen genes showed a difference of more than 200 bp and up to 900 bp, whereas the length difference for 443 proteins was less than 200 bp. Between 5U^u^ and 5D, OrthoFinder detected 615 single-copy orthologs, 286 of which showed the same number of exons, where 93 were of equal length. For 40 of these genes, the length difference was less than 200bp, while the difference for the remaining 53 genes ranged from 200 to 900bp. For the single-copy orthologs gene length varied significantly between the two genomes, ranging from 2 bp to 7 Kb.

**Table 4:** Gene structure comparison of *Ae. geniculata* (5M^g^) and *Ae. umbellulata* (5U^u^) chromosomes with group 5 chromosomes of Chinese spring.

Gene features	*Ae. geniculata* (5M^g^)	*Ae. umbellulata* (5U^u^)	CS (chr5A)	CS (chr5B)	CS (chr5D)
**Number of full length gene**	5100	2592	5429	5576	5563
**Total number of exons**	16885	6716	25836	26364	26015
**Total number of introns**	11129	3905	20387	20762	20437
**Number of single exon gene**	1836	1279	1316	1361	1392
**Mean exons per mRNA**	3.3	2.6	4.8	4.7	4.7
**Mean introns per mRNA**	2.2	1.5	3.8	3.7	3.7
**Total gene length (bp)**	12473015	4579550	18449098	19962808	18985249
**Total exon length (bp)**	6911779	2977095	8372686	8984820	8953505
**Total intron length (bp)**	4695503	1378321	9970751	10884969	9908071
**Mean gene length (bp)**	2445	1766	3398	3580	3412
**Mean exon length (bp)**	409	443	324	340	344
**Mean intron length (bp)**	421	352	489	524	484
**Length of longest gene (bp)**	22527	22543	47936	63855	58200
**Length of longest exon (bp)**	4322	3853	10983	6448	7292
**Length of longest intron (bp)**	7380	5261	38985	34008	44713
**Length of shortest gene (bp)**	351	325	108	105	105
**Length of shortest exon (bp)**	5	7	1	1	1
**Length of shortest intron (bp)**	60	60	2	2	2

The similarity percentage between the orthologs varies from 25 to 100%. More than 90 percent similarity was observed for 69% and 57% of genes in 5M^g^ and 5U^u^ with the CS genes, respectively.

In comparison to the homoeologous genes of CS for 5M^g^, 753 genes showed shorter transcript length and 692 showed longer transcript length, whereas in 5U^u^, 398 genes showed shorter, and 252 genes showed longer transcript length. Among the single exon genes, 127 genes were shorter, and 161 genes were longer in 5M^g^ whereas, in 5U^u^ 132 genes showed shorter and 520 genes showed larger genes length compared to CS homologs. For multi-exon genes, more genes were found with comparatively smaller genes length (5M^g^: 625, 5U^u^:255) compared to genes with larger lengths (5M^g^: 520 higher, 5U^u^: 167 higher) in both the studied chromosomes with respect to CS genes (**Table S3**).

**Figure 4:**
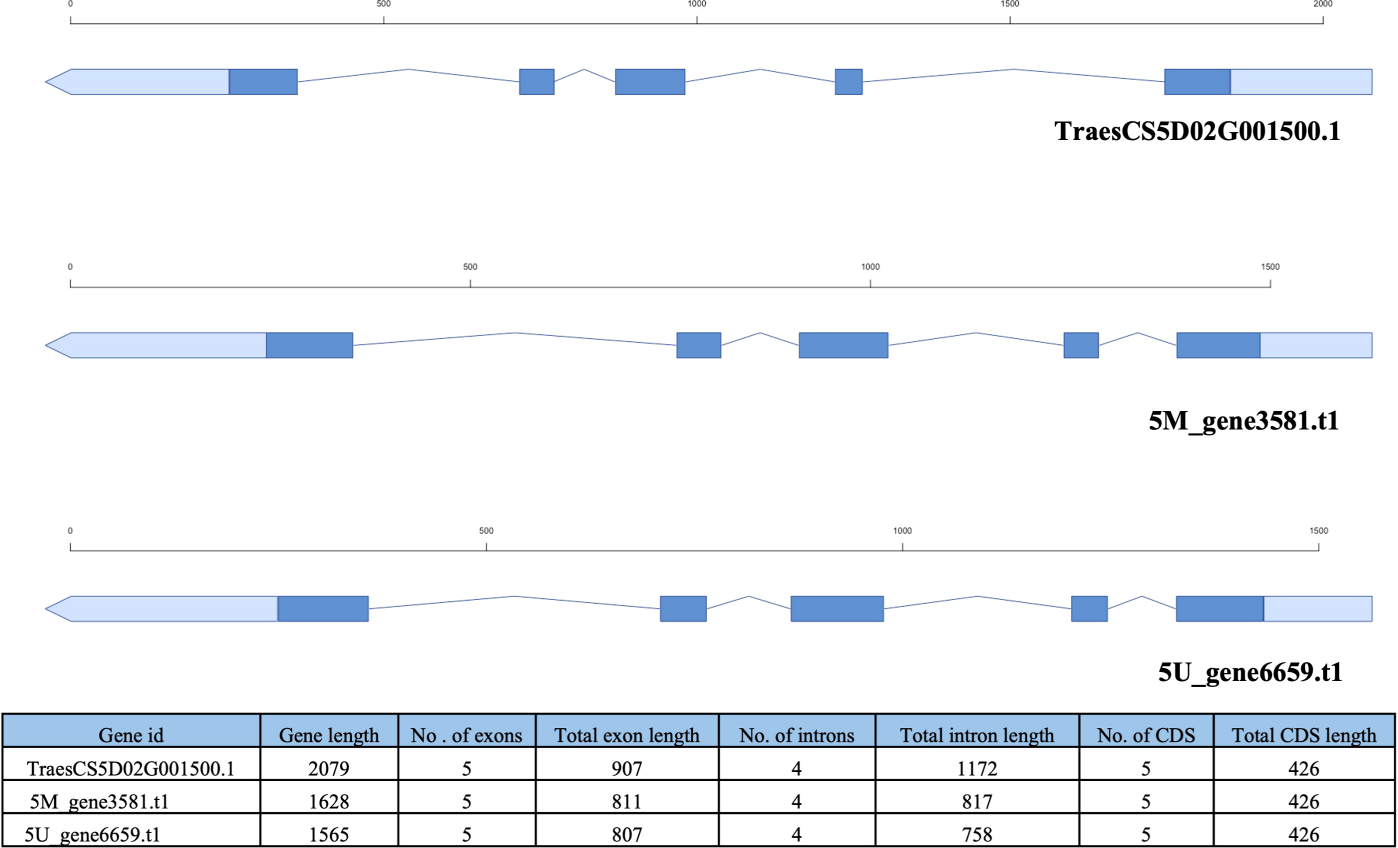
Comparison of gene structure in Chinese Spring, *Ae. geniculata* and *Ae. umbellultata*.

Out of 5,100 genes, a total of 4,031 genes were mapped on 5D using exonerate. Similarity-based filtering selected 2,355 genes, out of which finally 1,946 genes were used for analysis after removing erroneous alignments of larger than 15kb. For 1,673 genes, the number of exons in 5M^g^ matched exactly with the predicted number of exons from exonerate mapping. About 1,017 genes showed an equal number of exons between the exonerate mapping result and CS gene annotations. Out of these, 524 genes showed longer gene lengths in 5M^g^ and 493 demonstrated higher gene length in CS (**Table S4**). Concordance of the results was observed for 1,426 genes from similarity search and exonerate mapping results out of 1,446 single-copy genes compared. Although our results were based on gene length comparison of single copy-orthologs in a single chromosome study, these results indicate differences in gene structure between cultivated wheat and distinct wild relatives (**Figure 4**).

### Phylogenetic analysis

**Figure 5:**
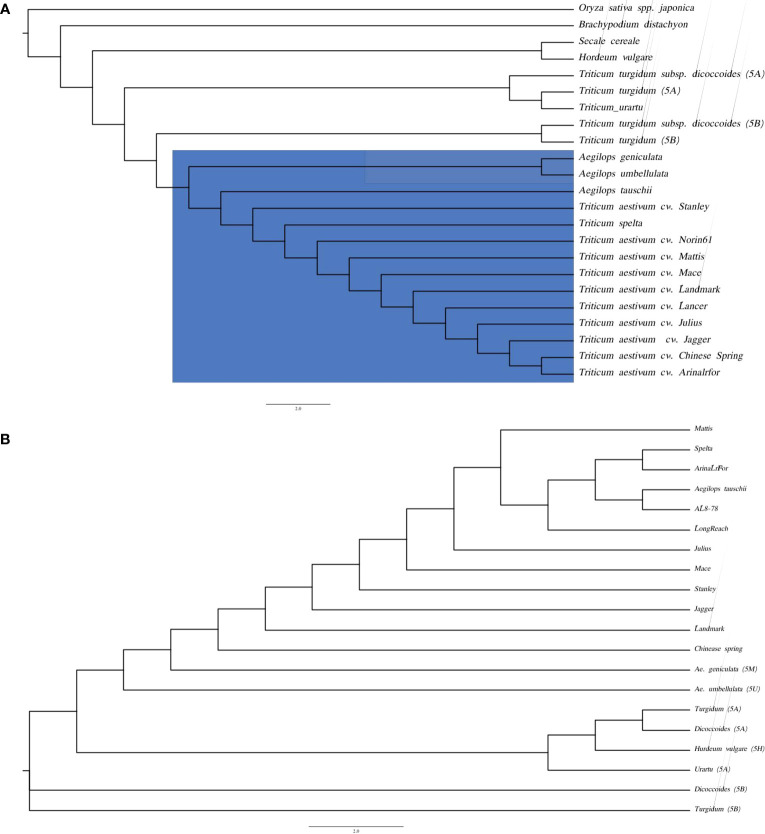
Phylogenetic trees based on homologous sequence **(A)** species tree of *Ae. geniculata* (5M^g^) and *Ae. umbellulata* (5U^u^) with other monocots genomes. **(B)** Kmer based species tree of wheat relatives.

Species tree based on the conserved gene alignments of the species i.e., *Oryza sativa* spp. japonica, *Brachypodium distachyon*, *Hordeum vulgare*, *Secale cereale*, *T. urartu*, *T. turgidum*, *T. turgidum* ssp. *dicoccoides*, *Ae. tauschii*, D genome of hexaploid wheat species (*T. aestivum* cv. Chinese Spring, *T. aestivum* cv. Arinalrfor, *T. aestivum* cv. Jagger, *T. aestivum* cv. Julius, *T. aestivum* cv. Lancer, *T. aestivum* cv. Landmark, *T. aestivum* cv. Mace, *T. aestivum* cv. Mattis, *T. aestivum* cv. Norin61, *T. aestivum* cv. Stanley, *T. spelta)* demonstrated close clustering of *Ae. genuculata* and *Ae. umbellulata* with the D genome group (**Figure 5A**).

Genes are the most conserved elements during evolution compared to the intergenic regions and serve as the main driving force for species evolution. Intergenic regions consist of repetitive elements and evolve at different rates compared to their genic counterparts. In the absence of the whole genome sequence, we performed the chromosome alignment of 5M^g^ and 5U^u^ with the group 5D chromosome of different wheat cultivars from the 10+ wheat genome project (Walkowiak et al., 2020). The Kmer-based phylogenetic tree reveals the closeness of the M and U chromosomes to the D group of chromosomes (**Figure 5B**). The k-mer-based tree also provides more resolution separating the M and U chromosomes at branches. There was a positive correlation between the phylogenetic tree and whole-genome alignments of the 5M^g^ and 5U^u^ reads to the wheat genome, where a higher percentage of read mapping was observed for the A genome compared to that of the B genome. These two observations support the closeness between M/U and A genomes.

### Evolution of gene families

**Figure 6:**
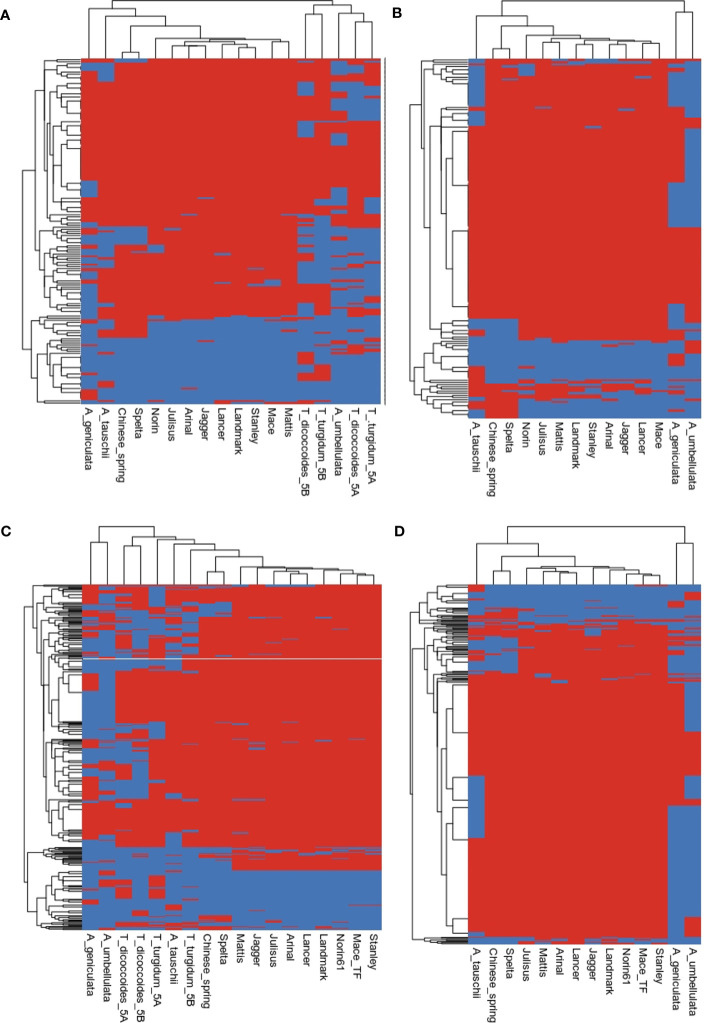
Heatmap inferring the presence (red) and absence (blue) of gene families of **(A, B)** resistance genes, **(C, D)** transcription factors.

The evolution of gene families shaped the present genomic landscape. We examined the evolution of resistance genes and transcription factors in the 5M^g^ and 5U^u^ chromosomes. Gene gain and loss events can be inferred based on the presence/absence of orthologs in the clades. It is believed that these events reflect origins or the loss of key characteristics within those clades (Natsidis et al., 2021). **Figure 6A** shows the heatmap of the presence/absence of R genes. In the presence of A and B genomes of tetraploid species, R genes formed two distinct clusters, where the *Ae. umbellulata* profile clustered with the A group of chromosomes and *Ae. geniculata* clustered with the D group of chromosomes (**Figure 6B**). In the absence of a tetraploid genome, *Ae. geniculata* and *Ae. umbellulata* together formed a group distinct from wheat and *Ae. tauschii*. In the case of transcription factors, *Ae. geniculata* and *Ae. umbellulata* formed a separate group from the rest of the studied genomes. The TF profile of 5M^g^ and 5U^u^ chromosomes was more similar to the tetraploid genome. A comparison of only the D-group of chromosomes also showed a distinct cluster for these genomes, where *Ae. tauschii* branched out separately (**Figures 6C, D**).

We compared the gene evolution between *Ae. geniculata*, *Ae. umbellulata*, *T. aestivum* cv. Chinese Spring and *Ae. tauschii.* Expansion of the TF gene families was observed on all the chromosomes. **Figure S4** displays expanded and rapidly evolving TF families. Nealy 16 and 36 TF gene families showed expansion in *Ae. umbellulata* and *Ae. geniculata*, respectively. For the R genes, 12 and 8 gene families showed expansion, whereas 12 and 4 gene families were found to be rapidly evolving in *Ae. geniculata* and *Ae. umbellulata*, respectively. The contraction was observed for two of the gene families.

### Genes involved in ETI and PTI response

**Figure 7:**
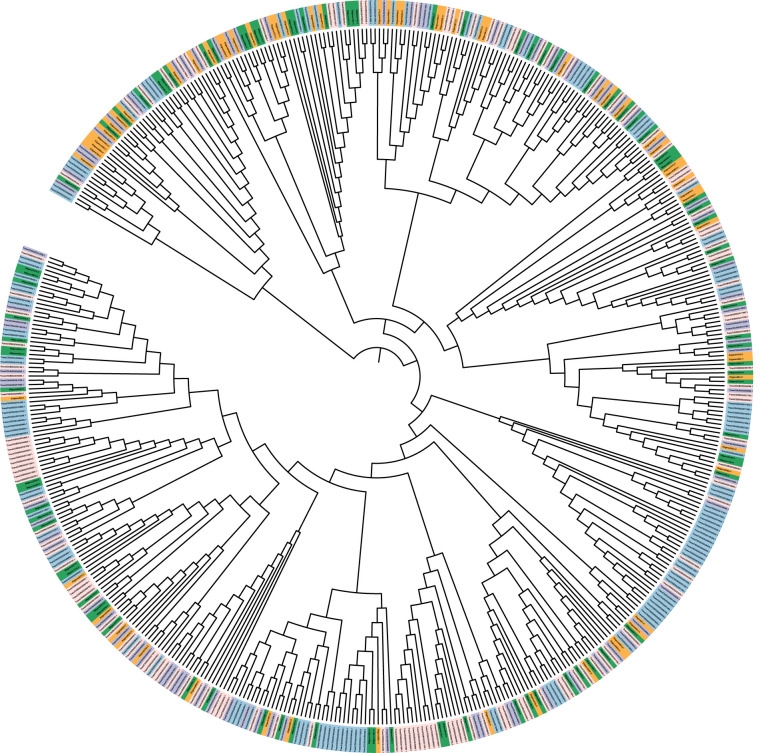
Mapman-based phylogenetic tree of genes involved in ETI and PTI response and resistance in *Ae. geniculata* (5M^g:^ green) and *Ae. umbellulata* (5U^u^: orange), 5A(purple),5B (pink), and 5D (blue) chromosomes of Chinese spring.

Mercator4 was used to assign MapMan functional annotations and pathways to *Ae. geniculata, Ae. umbellulata*, chr5A, chr5B, and chr5D. Genes involved in response to biotic stress for effector-triggered immunity (ETI) and pattern-triggered immunity (PTI) are shown in **Table 5**. These classifications are important as these will help us in categorizing the gene in response to pathogen attacks based on their similarity to known genes in Arabidopsis and rice. Chromosome 5D was found to be more enriched with pathogenicity-related genes compared to other chromosomes, owing its origin to *Ae. tauschii.* Comparatively fewer genes were reported for 5M^g^ and 5U^u^ chromosomes, while these chromosomes are well known to be a rich source of resistance genes. Chromosomes 5B and 5D displayed more genes with the “putative disease resistance protein” annotation compared to 5M^g^ and 5U^u^. A possible explanation may be the presence of new sources of resistance in these two genomes, while most of the genes in CS may have lost their functional significance with sequence divergence. This requires further exploration through whole genome studies. **Figure 7** displays the putative genes involved in pathogen response to ETI and PTI response along with putative resistance genes.

**Table 5:** Mapman based assignment of functional gene categories of effector-triggered immunity (ETI) and pattern-triggered immunity (PTI) response.

Class	Category	Subcategory	5M^g^	5U^u^	5A	5B	5D
Pattern-triggered immunity (PTI)	Nematode elicitor response	receptor protein kinase (NILR)	0	0	0	0	2
	Fungal elicitor response	protein kinase (PBL27/RLCK185)	0	0	0	0	3
	Fungal elicitor response	chitin receptor protein kinase (CEBiP)	1	2	2	1	0
	Bacterial elicitor response	protein kinase (PCRK)	0	0	0	0	1
		FLS2-BAK1 flagellin receptor complex.flagellin receptor protein kinase component (FLS2)	0	0	0	0	1
		FLS2-BAK1 flagellin receptor complex.dynamically associated protein kinase (BIK1)	1	0	1	1	0
		FLS2-BAK1 flagellin receptor complex.co-receptor kinase component (BAK1)	0	0	0	0	1
	network.bacterial elicitor response.protein kinase (BSK1)	network.bacterial elicitor response.protein kinase (BSK1)’	0	0	1	0	0
Effector-triggered immunity (ETI)	TNL-mediated effector-triggered immunity	regulatory protein (ADR)	1	0	1	1	0
		posttranscriptional regulatory protein (FPA)	1	0	1	1	0
	RIN4-RPM1 immune signalling	regulatory factor (RIN4) guarded by RPM1/RPS2 activities	1	0	0	1	1
		CC-NLR-type effector receptor (RPM1)	0	0	0	0	1
	regulatory protein (RAR1)	regulatory protein (RAR1)	0	0	0	0	1
	immunity suppressor (SOBER/TIPSY)	immunity suppressor (SOBER/TIPSY)	0	0	0	0	3
	network regulatory protein (EIJ1)	network regulatory protein (EIJ1)	0	0	1	1	1
	EDS1-PAD4/SAG101 signalling heterodimers	component EDS1	1	1	1	1	0
Systematic acquired resistance	regulatory protein (CBP60/SARD)	regulatory protein (CBP60/SARD)	5	1	7	9	1
	pipecolic acid metabolism	pipecolate oxidase (SOX)	4	2	3	4	0
	NPR1-interactive transcription factor (TGA)	NPR1-interactive transcription factor (TGA)	0	0	0	0	1
	pathogen polygalacturonase inhibitor (PGIP)	pathogen polygalacturonase inhibitor (PGIP)	2	1	1	1	3
	defensin activities.defensin (PDF2)	defensin (PDF2)	1	2	4	1	3
Unassigned	not assigned.annotated	not assigned.annotated	1	5	2	0	0

### Comparative analysis of 5M^g^ and 5U^u^ introgressions providing resistance against leaf rust using transcriptome data under leaf rust stress

**Figure 8:**
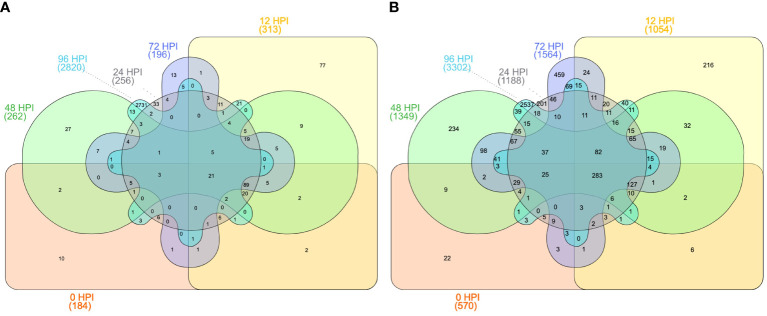
Differentially expressed genes under different time intervals **(A)** between T756 and WL711 and **(B)** between T598 and WL711 at 6-time intervals after inoculation: 0 (hours post inoculation: HPI), 12 HPI, 24 HPI, 48 HPI, 72 HPI, and 96 HPI.

To compare the gene expression between the homoeologous genes from 5M^g^, 5U^u,^ and wheat, we used highly specific translocation germplasm available to us (Tiwari et al., 2014; Tiwari et al., 2015; Yadav et al., 2016; Bansal et al., 2020; Steadham et al., 2021). Using these resources, we performed differential expression analysis to determine potential candidate genes translocated from 5M^g^ of *Ae. geniculata* to the terminal distal region (10Mb) on the 5D chromosome of wheat using two translocation lines T756 & T598 (pau16052 & pau16055) challenged with leaf rust inoculum. Differential expression analysis resulted in 3,196 differentially expressed genes (DEGs) between T756 and WL711, and 5,099 DEGs between T598 and WL711. A total of 6,512 genes were differentially expressed in two ILs and 1,784 genes were common between both studies. T756 showed 2,058 up-regulated and 1,138 down-regulated genes, while T598 displayed 2,833 up-regulated and 2,265 down-regulated genes (**Tables 6**, **S5**). DEGs at different HPIs intervals between T756 and WL711, and T598 and WL711 are shown in **Figure 8**. We found that 21 genes in T756 and 283 genes in T598 were shared among all HPI intervals. In both the ILs, catalytic activity, and transferase activity were found with the higher number of up-regulated genes, while DNA binding and biological regulation exhibited down-regulation. Response to the biotic stimulus was up-regulated in T756, while no corresponding GO term was detected in T598. A total of 283 genes were shared at different HPI in T598 compared to only 21 shared genes in T756.

**Table 6:** Differentially expressed genes in introgression lines T756 and T598 in comparison to WL711.

HPI interval	Total DE gene	DEG (FDR<0.001)	Upregulated	Down regulated
T756 (WL711+Lr57)				
0HPI	32052	184	101	83
12HPI	36214	313	187	126
24HPI	34150	256	136	120
48HPI	34406	262	150	112
72HPI	34685	196	110	86
96HPI	40457	2820	1847	973
T598 (WL711+Lr57)				
0HPI	33610	570	344	226
12HPI	36681	1054	691	363
24HPI	35529	1188	670	518
48HPI	35597	1349	866	483
72HPI	36242	1564	943	621
96HPI	40799	3302	1713	1589

The gene ontology (GO) plot using WEGO indicated a higher number of genes involved in binding and catalytic activity under molecular functions, whereas biological processes showed higher number of genes under metabolic and cellular processes followed by a response to stimulus process in both introgression lines (**Figure S5**). Gene ontology enrichment using goseq demonstrated a higher percentage of genes being involved in ATP binding, ADP binding, and protein kinase activity under the molecular function category playing a significant role in the signaling process (**Figure S6**). Genes belonging to chitinase activity were also found enriched. These genes may play a role in degrading the membrane-bound carbohydrate structures on the fungal cell wall. Monooxygenase and oxidoreductase activity was also found to be enriched under defense response. In biological processes, protein phosphorylation and defense response categories were prevalent in both the introgression lines. In T756, the integral component of membrane was found enriched under cellular component, while the same was not detected in T598.

Nearly 417 genes were found differentially expressed on chromosome 5D collectively in both ILs, with 78 up-regulated and 53 down-regulated genes in T756; 95 up-regulated and 267 down-regulated genes in T598. We filtered DEGs in the 10 Mb distal region of chromosome 5DS, with reported introgression for Lr57/Yr40 (Kuraparthy et al., 2007; Yadav et al., 2016; Steadham et al., 2021). Out of 173 high-confidence genes in the region, 54 genes were found to be differentially expressed. Most of the genes were downregulated in the candidate region with prominent transferase and dephosphorylation activity shown in **Figure S5**. *Ae. geniculata* is a member of the tertiary gene pool and there are chances that homologous genes may not be present on the CS designated chromosomes. We looked for the orphan genes present on unknown chromosomes of CS. On the unanchored chromosome, there were 191 DEGs, including 146 genes for T598 and 90 genes for T756. We searched for the genes with protein kinase, NB-ARC, or LRR domains within these DEGs and discovered 26 genes. There were four different genes in each IL, which were up-regulated in at least four HPI intervals. These genes appeared to be potential candidates for the Lr57 because they were significantly upregulated, with expression changes ranging from 2 to 9-fold (**Table S5**). We looked for the homologs of these genes on our 5M^g^ assembly, but blast results were not significant. These genes may be the source of resistance with higher up-regulation, but it is difficult to establish their origin from *Ae. geniculata.* The syntenic map of the 10Mb introgressed region created using the contigs of the mapped genes is shown in **Figure S8**.

### Development of SSR and SNP markers

The MISA program detected 47,358 and 28,612 SSRs in 5M^g^ and 5U^u^ chromosomes, respectively. The majority of the SSRs were either monomeric or dimeric types (**Figure S7**). Compound SSR constituted 10% and 8% of the total SSRs in 5M^g^ and 5U^u^, respectively. Primers were designed for 31,750 and 13,199 SSRs for the two chromosomes, respectively. To identify genome-specific primers, we mapped the 5M^g^ primer pairs to 5U^u^ and 5D, similarly, 5U^u^ primer pairs were mapped to 5M^g^ and 5D. With the stringent criteria of two mismatches and zero gaps, 4,051 and 2,389 primers of 5M^g^ mapped to 5D and 5U^u^, respectively. In 5U^u^, 1,672 and 1,675 uniquely mapped primer pairs were detected for 5D and 5M^g^, respectively. Chromosome 5D and 5M^g^ shared 1,044 primer pairs from 5M^g^, while 5D and 5M^g^ shared 884 primer pairs for 5U^u^ (**Table S7**). Kompetitive allele specific PCR (KASP) markers were developed from the homozygous SNPs between 5M^g^ and 5D , and 5U^u^ and 5D (**Table S8**).

## Discussion

Characterization of genetic diversity underlying a phenotype is critical in improving wheat against several biotic and abiotic stresses. Polyploidization resulted in enhanced adaptability of the wheat to diverse environments outside its center of origin. At the same time, domestication and selective breeding for high yield have reduced the genetic base of cultivated wheat varieties (Feuillet et al., 2008). Wild and distant relatives of wheat contain rich genetic diversity for its improvement and many of these wild relatives are well-documented to provide resistance to biotic and abiotic stresses. *Ae. geniculata* and *Ae. umbelulata* are two such species from the tertiary gene pool of wheat and harbor several genes and alleles for wheat improvement. There is consensus about the conservation of large scale synteny of the homoeologous genes and chromosomes, or chromosomal regions based on available genomics datasets, and comparative genome analysis of wheat’s wild relatives (Walkowiak et al., 2020). Whole genome sequencing of *Ae. comosa* and *Ae. umbellulata* demonstrated the collinearity of the M and U genome with the D genome (Said et al., 2021). Our initial results have indicated that the 5M^g^ chromosome is homoeologous to wheat chromosome 5D. Previous analysis of translocations lines TA5602 (5% translocation of 5M^g^ short arm on 5D of wheat) and pau3732 (5% translocation of 5U^u^ short arm on 5D of wheat) showed no major rearrangements in the introgressed 9.5 Mb region (Bansal et al., 2017; Steadham et al., 2021). These lines and available datasets from shotgun sequencing of chromosomes 5U^u^, 5M^g^, and RNAseq results from translocation lines and availability of reference genomes of wheat lines, provide us an excellent opportunity to look at the evolutionary patterns in wild and domesticated homoeologous accessions, especially for the disease resistance genes in targeted physical regions.

In the present study, we reported 397 Mb and 243 Mb assembled sequences of flow-sorted chromosomes 5M^g^ and 5U^u^ of *Ae. geniculata* and *Ae. umbellulata* retrieved from public sources to study the pattern of gene evolution in group 5 chromosomes. An N50 of 3.4kb and 1.13 kb was observed for both 5M^g^ and 5U^u^ chromosomes. Assembled sequence demonstrated a repeat content of 79% and 63%, with 736 and 383 conserved BUSCO genes for 5M^g^ and 5U^u^, respectively. The N50 achieved was higher than that of the previous study for 5M^g^ (1.1kb) (Tiwari et al., 2015), but was lower for 5U^u^ (2.9kb) (Bansal et al., 2020). Due to the fragmented assembly and partial genes, we restricted our analysis to the full-length genes carrying both transcription start and termination sites, which still give us access to more genes than the previous studies. The full-length genes reported showed sequence homologs for 84% and 54% of sequences in 5M^g^ and 5U^u^ in the NCBI non-redundant database. Functional annotations (protein domain, enzyme classes, and GO annotations) were reported for more than 50% of these genes. The majority of TFs identified belong to C2H2, bHLH, B3, MYB-related, bZIP, M-type MADS, WRKY, and ERF classes and constitute 67% and 60% of the TF classes in 5M^g^ and 5U^u^, respectively. C2H2 zinc finger transcription factors play important roles in plant growth, development, and biotic and abiotic stress resistance (Han et al., 2020). Similarly, bHLH plays a significant role in drought, salt, and chilling stress (Sun et al., 2018). MYB transcription factors are known for their role in plant development, secondary metabolism, hormone signal transduction, disease resistance, and abiotic stress tolerance (Katiyar et al., 2012). Basic leucine zipper (bZIP) gene family is one of the largest transcription factor families in plants, and members of this family play important roles in multiple biological processes such as light signaling, seed maturation, flower development as well as abiotic and biotic stress responses (Wang et al., 2018). MADS-box transcription factors were found to be involved in floral organ identity determination (Theißen et al., 2016). ERF transcription factors play many diverse functions in cellular processes, such as hormonal signal transduction, response to biotic and abiotic stresses, regulation of metabolism, and in developmental processes in many plant species (Nakano et al., 2006). The richness of these TFs classes makes 5M^g^ and 5U^u^ chromosomes an attractive target for introgressions, also evident from the previous introgression studies. In the present study, both the chromosomes displayed higher number of resistance genes.

Despite the restricted growth environment for *Ae. umbellulata*, it showed higher genetic diversity than *Ae. tauschii* (Okada et al., 2018b). According to Stoilova et. al, chromosomal substitution of 6U has been shown to provide resistance to powdery mildew at both the seedling and adult plant stages (Stoilova and Spetsov, 2006). Synthetic lines generated through crossing between durum and *Ae. umbellulata* showed increased plant height and the number of spikes and a decrease in spike length (Okada et al., 2020). Grain hardness is regulated by the Hardness (Ha) locus on 5D, which contains the genes Pina-D1 and Pinb-D1 that code for puroindoline proteins. For hard-textured common wheat, variations in *Ae. umbellulata* is considered a useful resource for enlarging the diversity of grain hardness (Okada et al., 2018a). Both the ILs used in the present study have higher grain hardness than the recipient parent (data not presented). Wheat-*Ae. umbellulata* introgression carrying 1U^u^ demonstrated accumulation of glutenin macropolymer (GMP) compared to the recurrent parent Chinese Spring (Du et al., 2019), implying its potential application for improving the end-use property of the wheat flour.

During evolution, gene order or collinearity tends to be conserved across species (Huynen and Bork, 1998). Syntenic block represents the regions with shared homologous genes between the two genomes derived from the common ancestor (Tang et al., 2011). These blocks are often used to investigate gene evolution that might have shaped the speciation. Natural or artificial plant hybridizations typically result in the addition of new gene combinations that enhance the genic density of the available germplasm (Arrigo et al., 2011). Ramírez-González et al. demonstrated the biased expression in nearly 30% of homoeologous genes in wheat (Ramírez-González et al., 2018). DEGs from RNA expression analysis of susceptible and resistant introgression lines provided an array of genes involved in resistance belonging to classes of DNA binding, catalytic activity, and protein kinase activity. Chitinase-related genes were also found to be enriched in DEGs. DEGs in the reported introgressed region were found to be down-regulated. Four genes belonging to resistance gene class were found to be highly expressed at most of the HPI intervals. We were unable to detect their origin from 5M^g^ which may be due to fragmented assembly.

Evolutionary studies revealed the closeness between *Ae. geniculata* and *Ae. tauschii* (Middleton et al., 2014; Li et al., 2015). Chloroplast genome-based studies have shown the divergence of *Ae. tauschii* and *Ae. geniculata* nearly around 1.62 mya (Middleton et al., 2014). Li *et.al.*, derived distinct clusters for M, S, and D genomes using the chloroplast sequence of the progenitors (Li et al., 2015). A high number of 60% homoeologous genes were shared between 5M^g^,5U^u^, and group5 chromosomes (A, B, D genome) of wheat. There were nearly 514 single-copy orthologs attributed to a lower gene count of *Ae. umbellulata*. A comparison of the 5M^g^ and 5U^u^ with the *Ae. tauschii* and genomes of 10 other wheat cultivars is shown in **Figure 2B**. Comparison of 5M^g^ and 5U^u^ with the publicly available exome data of hexaploid and tetraploid wheat, positioned the two assembled chromosomes into a separate cluster, demonstrating the distinctness of the U and M genome from the D genome. We concluded that M and U genomes hold a phylogenetic position in between the A and D genomes. This was further supported by the read-mapping study, which showed a considerably higher number of read mapping on the A genome compared to the B genome. Although our observations were based on the single chromosome data, a purity of ~95% of flow-sorted chromosomes, reduces the possibility of contamination in both chromosomes, which come from different studies. But still, further exploration is needed in the presence of higher similarity to A genome.

Read mapping demonstrated some sort of similarity between 5M^g^ and chr1A, and 5U^u^ and chr2A. These sites can be considered the potential hotspot region for introgression or hybridization although no reported introgression was found in the support. A similar observation was depicted through the chromosome-level phylogeny, which grouped the M genome with the D genome and ordered these closer to the A genome. The likelihood of contamination from other chromosomes is reduced by the 95% purity of the flow-sorted chromosomes. Chromosome flow-sorting made a significant contribution in lowering the complexity of the wheat genome (Marcussen et al., 2014). Previously natural hybrids of *Ae. geniculata* and wheat have been reported to exist under field conditions in central Spain (Loureiro et al., 2006). However, given the increased similarity to the A genome, further genome-scale exploration is needed in the future. Mena et al. demonstrated 5A/5M^v^ introgression of *Ae. ventricosa* in the wheat-Aegilops system. (Mena et al., 1993). Researchers have reported substitution lines where 7A and 7B were replaced by 7M providing resistance to Fusarium head blight (fhb), powdery mildew, and stripe rust (Yang et al., 2022). The significant number of mutations observed with the Chinese Spring provide distinction to the genomes. Indels accounted for one-fourth of the total variants, responsible for differences in the gene models between alien chromosomes and Chinese Spring. Nearly 90% and 80% of the genes on chr5D were impacted by SNP variants from 5M^g^ and 5U^u^, of which 35% were missense-type variants. A total of 4-5 percent of variants showed a moderate effect on the structure and function of the genes.

During the evolution, expansion in the gene sequence resulting from the incorporation of large insertional elements in the intronic region has been observed. Sela and colleagues demonstrated 60% of TE insertions within introns compared to the transcribed region within the mammalian genome (Sela et al., 2010). We observed a significant difference in gene length for the 5M^g^ and 5U^u^ genes compared to chr5D. Tertiary gene pool species displayed a shorter gene structure compared to their hexaploid counterpart, evidenced by the fewer intron per mRNA and smaller mean intron length. Differences were also observed in single-copy orthologs, where genes with the same number of exons and equal protein length showed differences in total gene length. Mapping of 5M^g^ genes on 5D chromosome resulted in similar gene structure, with a different exon-intron model (**Figure 4**).

Genome-wide AFLP markers have proved reliable to detect hybridization and introgression events. Natural hybridization in *wheat-Aegilops* species has been reported when present or grown in proximity (Arrigo et al., 2011). Whole genome/chromosome sequencing can be very helpful in detecting introgressions (Walkowiak et al., 2020). We observed traces of significant introgression on the telomeric region of the wheat chromosome 5D from 5M^g^ of *Ae. geniculata* spanning from 546 Mb to 566 Mb with the read coverage density nearly equal to 1 in a 1Mb window. The region consisted of 394 high confidence genes. However, no literature-based evidence was found for the introgression. The developed SSR markers can be used for detecting the present and other introgressions on chromosome 5D (**Table S3**).

## Conclusion

With their extensive genetic diversity, *Ae. geniculata* and *Ae. umbellulata* represent a valuable resource for wheat improvement. We detected a wide range of orthologs, both shared and species-specific, which can be used enhance disease resistance and quality traits. Both chromosomes harbor an abundance of transcription factors that might play a regulatory role in the pathways for tolerance to drought, salt, and chilling stresses. Our study highlighted the differences in the gene structure between the members of tertiary gene pool and cultivated wheat. These may be the results of incorporation of large TE insertions in wheat genome. Gene families in the tertiary gene pool are still under evolutionary pressure and are expanding. Phylogenetic and read-mapping studies demonstrated the higher resemblance of M and U genomes to the D genome, followed by A genome. The remarkably diverse set of TFs with a role in biotic and abiotic stress and resistance genes make these two chromosomes interesting targets for introgression studies.

## Data availability statement

The datasets presented in this study can be found in online repositories. Previously published accession number(s) can be found in the article. RNAseq data for the Ils (T598) have been deposited at National Center for Biotechnology Information (NCBI) BioProject: PRJNA988449.

## Author contributions

VT, IY, NR, CU, and PC developed the idea and planned the research, and generated resources. VT supervised the research, performed the experiments, and analyzed the data. SK generated the transcriptome datasets and provided edits. IY and VT wrote the manuscript with valuable edits from all co-authors. All authors contributed to the article and approved the submitted version.

## Supplementary material

Supplementary Figure 1 Conserved BUSCO-gene-based evaluation of *Ae. geniculata* (5M^g^) and *Ae. umbellulata* (5U^u^).

Supplementary Figure 2 Whole chromosome display of density of mapped reads of *Ae. geniculata* and *Ae. umbellulata* on chromosome 5D of Chinese spring.

Supplementary Figure 3 Coverage of mapped reads of *Ae. geniculata* (5M^g^) and *Ae. umbellulata* (5U) on the genic region of Chinese spring.

Supplementary Figure 4 Evolution of gene families of resistance gene and transcription identified using CAFÉ. **(A)** expansion of resistance genes, **(B)** contraction of resistance genes, **(C)** rapidly evolving resistance genes, **(D)** expansion of TFs, **(E)** contraction of TFs, and **(F)** rapidly evolving TFs genes. Numbers in the bracket indicate the gene number.

Supplementary Figure 5 Gene ontology classification of differentially expressed genes in **(A)** introgression line T756 and **(B)** introgression line T598.

Supplementary Figure 6 Gene ontology enrichment total differentially expressed genes **(A)** T756 and WL711 and **(B)** T598 and WL711.

Supplementary Figure 7 Distribution of simple sequence repeats in *Ae. geniculata* (5M^g^) and *Ae. umbellulata* (5U^u^).

Supplementary Figure 8 Synteny of *Ae. geniculata* (5M^g^) and *Ae. umbellulata* (5U^u^) chromosomes with chromosome 5D of Chinese spring in 10Mb introgression from introgression lines. Connecting lines represents the contig location on 5D.
