# Supplementary figures and images for: Comparative genomic analysis of 5Mg chromosome of Aegilops geniculata and 5Uu chromosome of Aegilops umbellulata reveal genic diversity in the tertiary gene pool
Source: Front Plant Sci. 2023 Jul 13;14:1144000. doi: 10.3389/fpls.2023.1144000 (PMC10373596; doi:10.3389/fpls.2023.1144000)

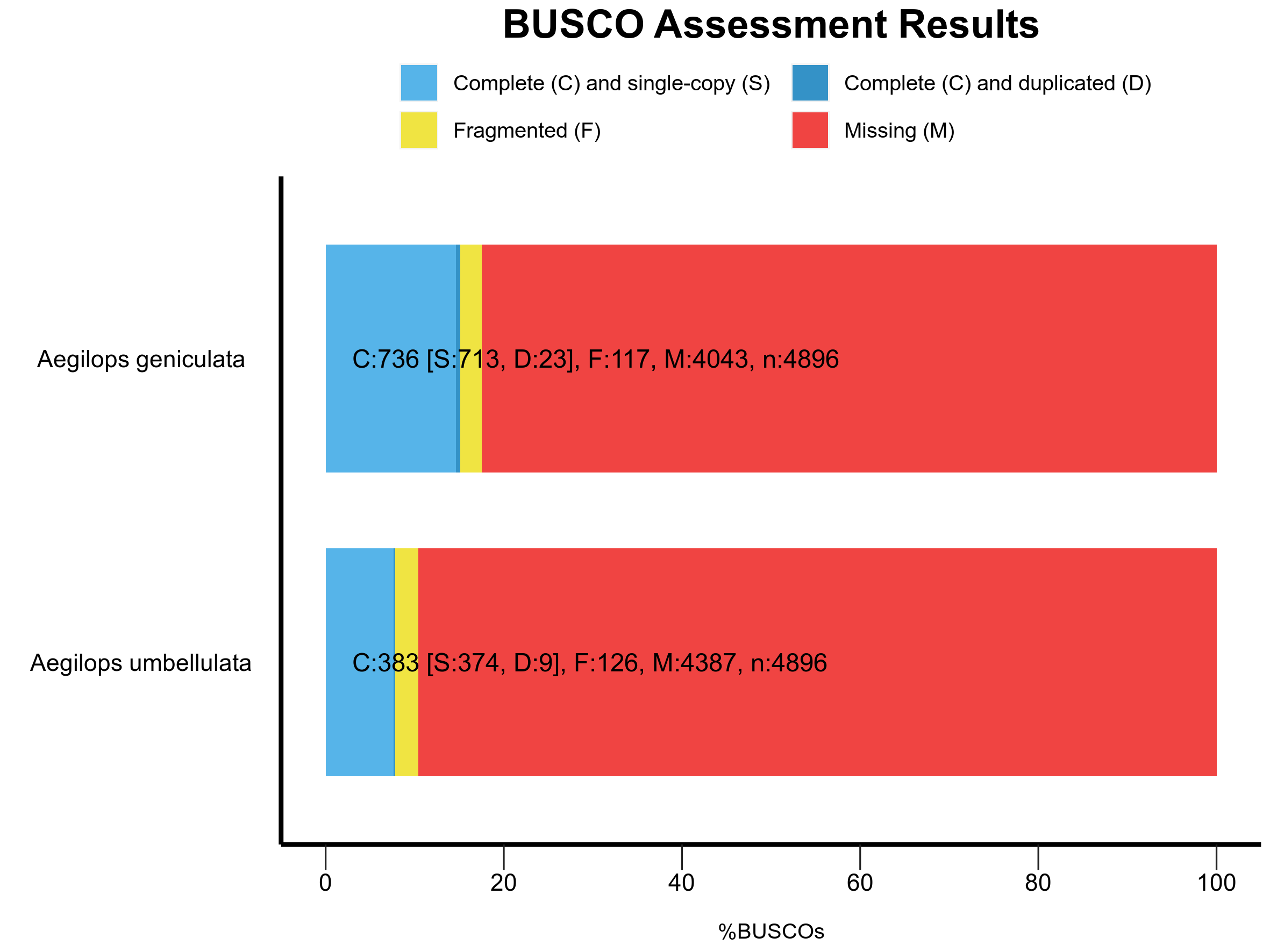

Supplement: Supplementary Figure 1 — Conserved BUSCO-gene-based evaluation of Ae. geniculata (5Mg) and Ae. umbellulata (5Uu). [file Image_1.png]

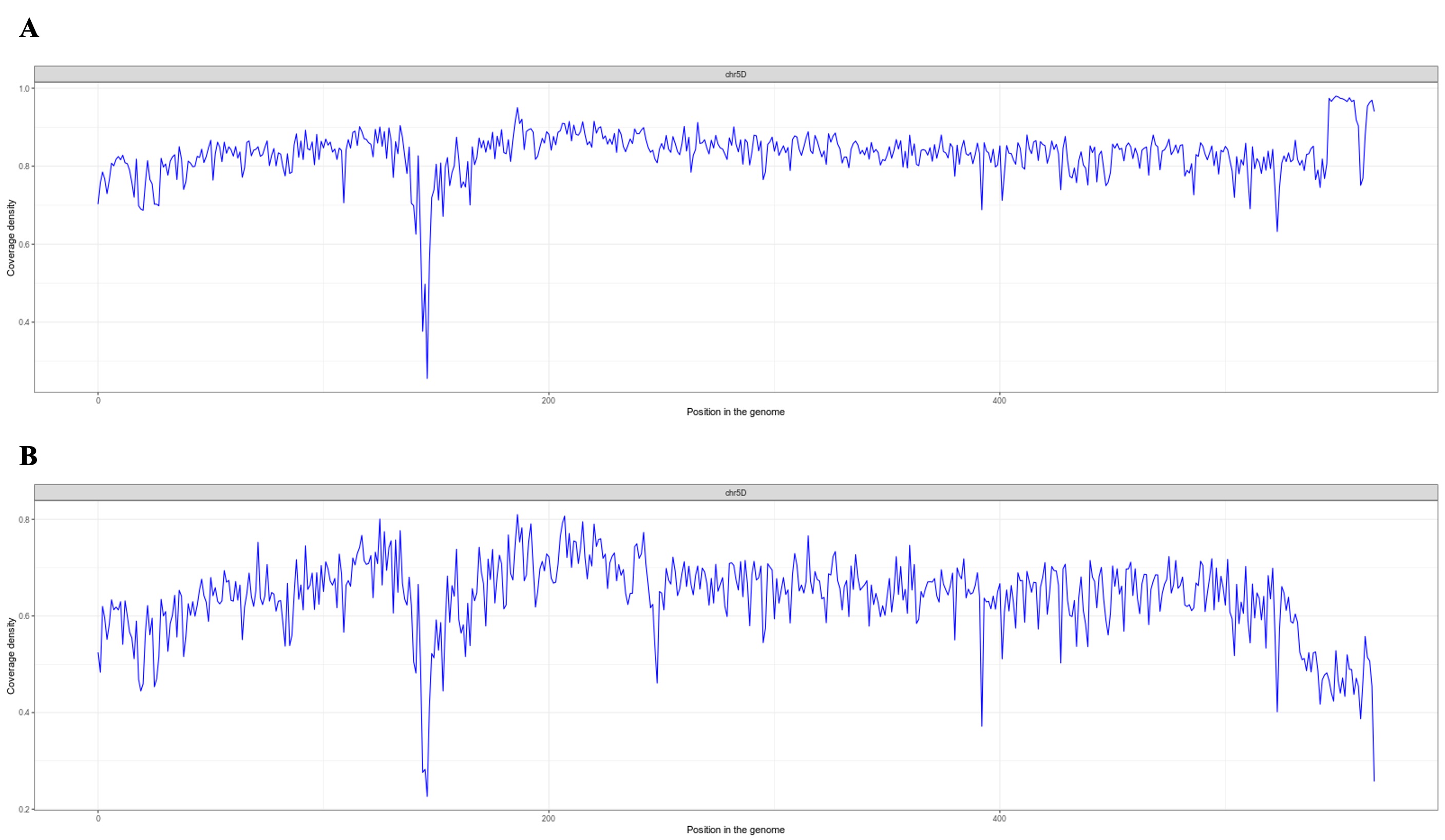

Supplement: Supplementary Figure 2 — Whole chromosome display of density of mapped reads of Ae. geniculata and Ae. umbellulata on chromosome 5D of Chinese spring. [file Image_2.jpeg]

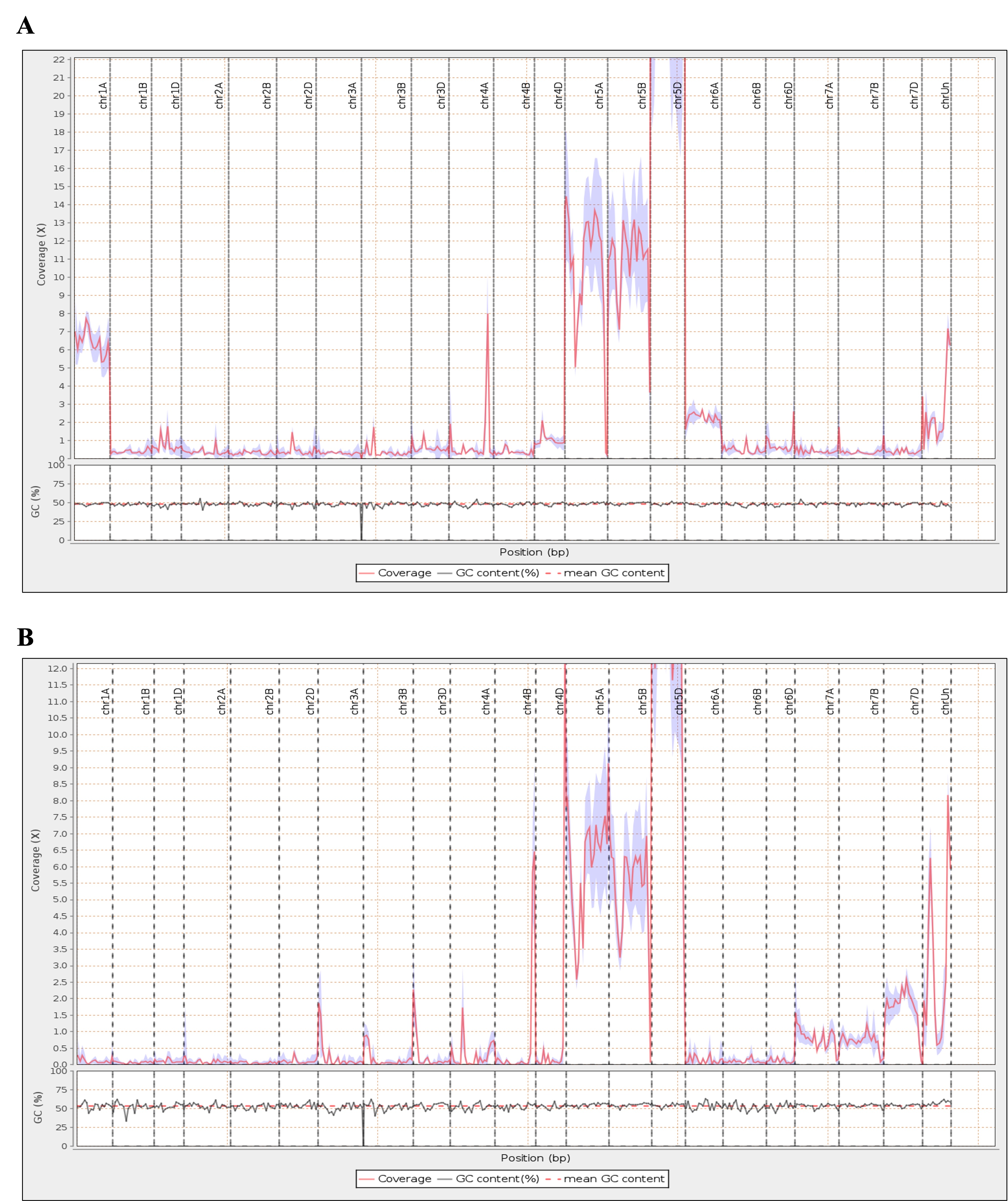

Supplement: Supplementary Figure 3 — Coverage of mapped reads of Ae. geniculata (5Mg) and Ae. umbellulata (5U) on the genic region of Chinese spring. [file Image_3.jpeg]

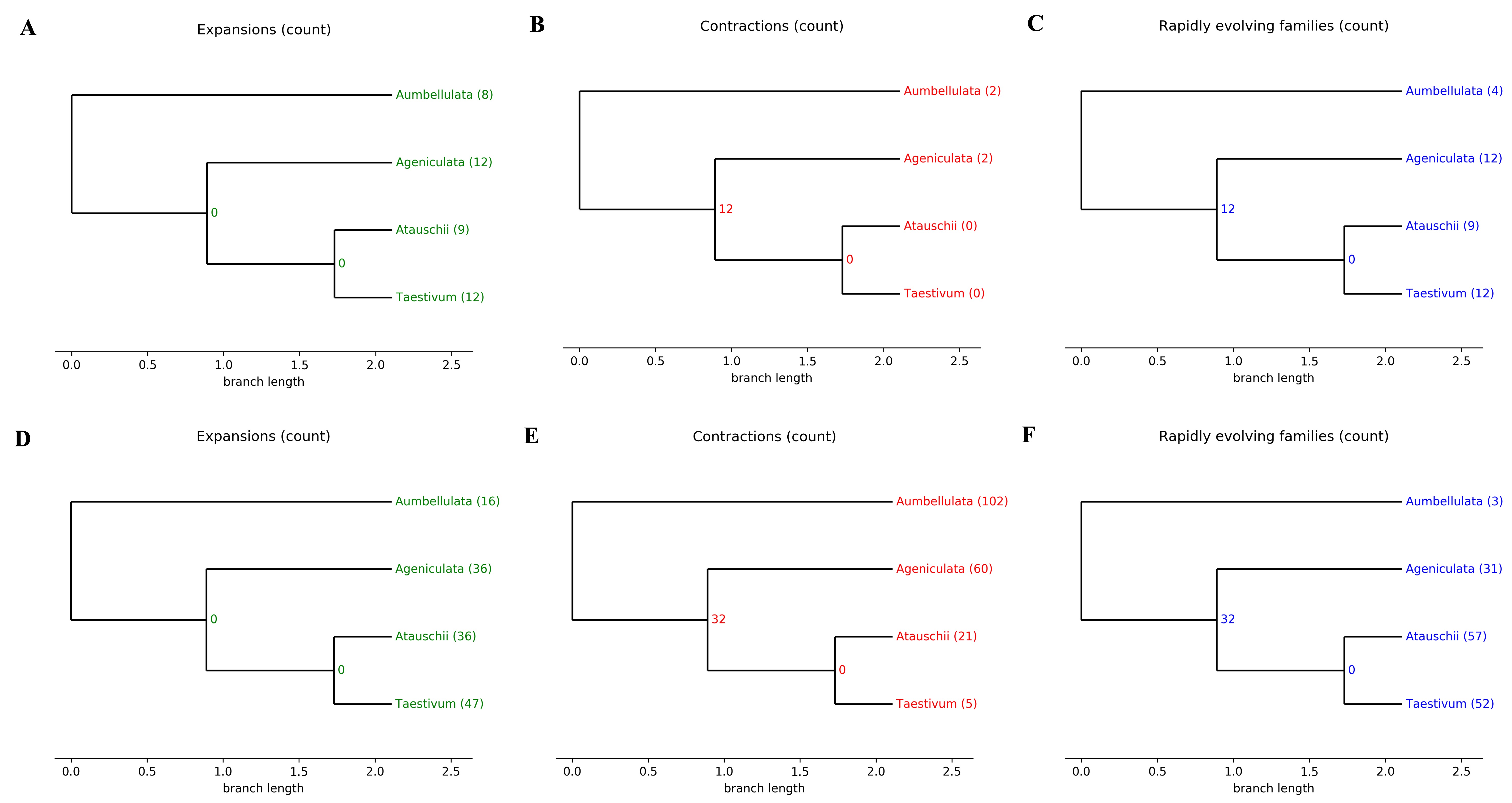

Supplement: Supplementary Figure 4 — Evolution of gene families of resistance gene and transcription identified using CAFÉ. (A) expansion of resistance genes, (B) contraction of resistance genes, (C) rapidly evolving resistance genes, (D) expansion of TFs, (E) contraction of TFs, and (F) rapidly evolving TFs genes. Numbers in the bracket indicate the gene number. [file Image_4.jpeg]

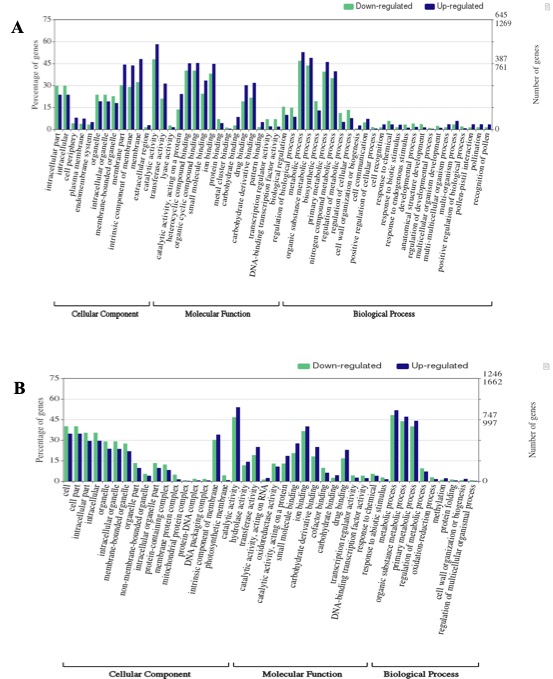

Supplement: Supplementary Figure 5 — Gene ontology classification of differentially expressed genes in (A) introgression line T756 and (B) introgression line T598. [file Image_5.jpeg]

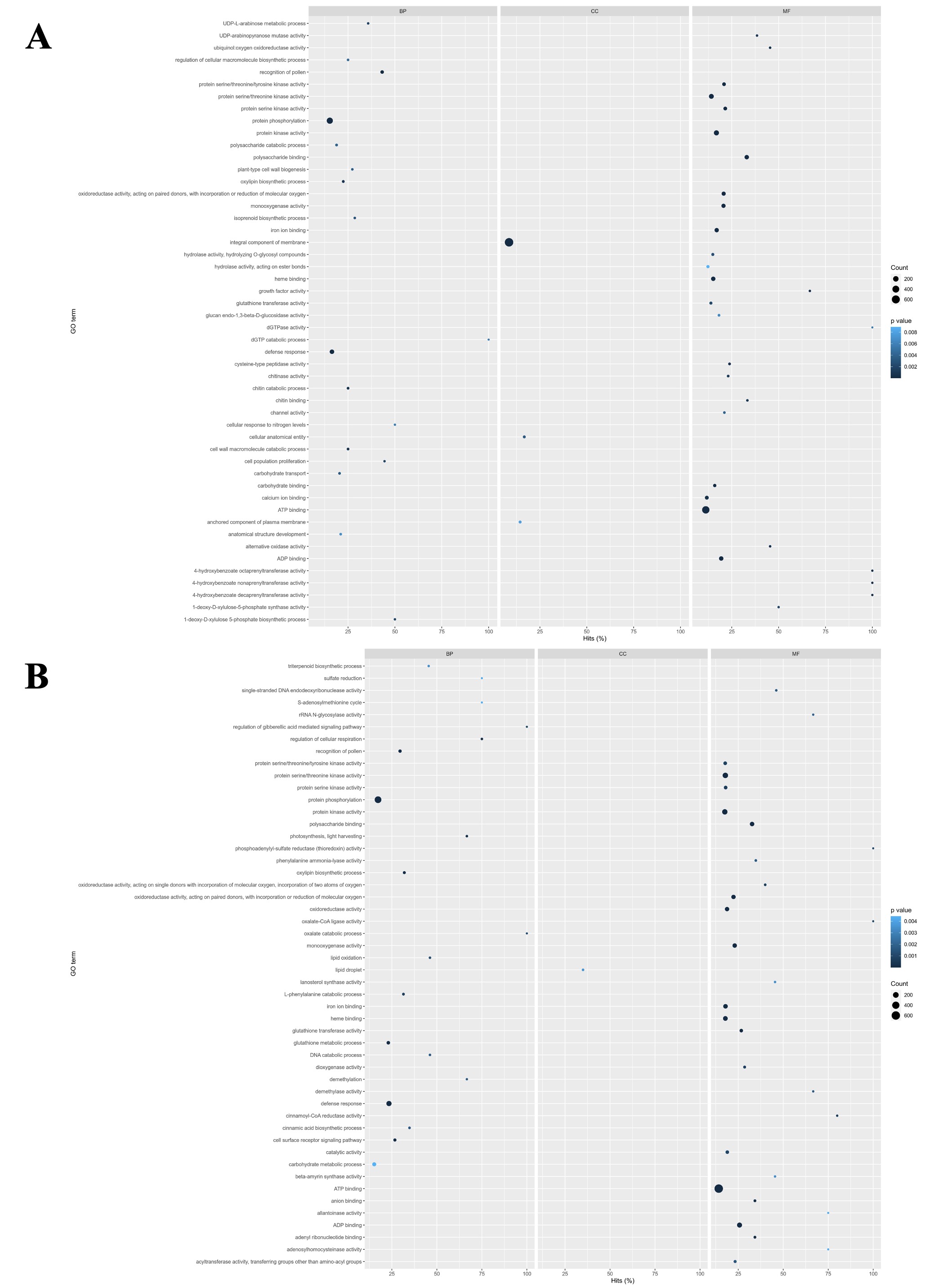

Supplement: Supplementary Figure 6 — Gene ontology enrichment total differentially expressed genes (A) T756 and WL711 and (B) T598 and WL711. [file Image_6.jpeg]

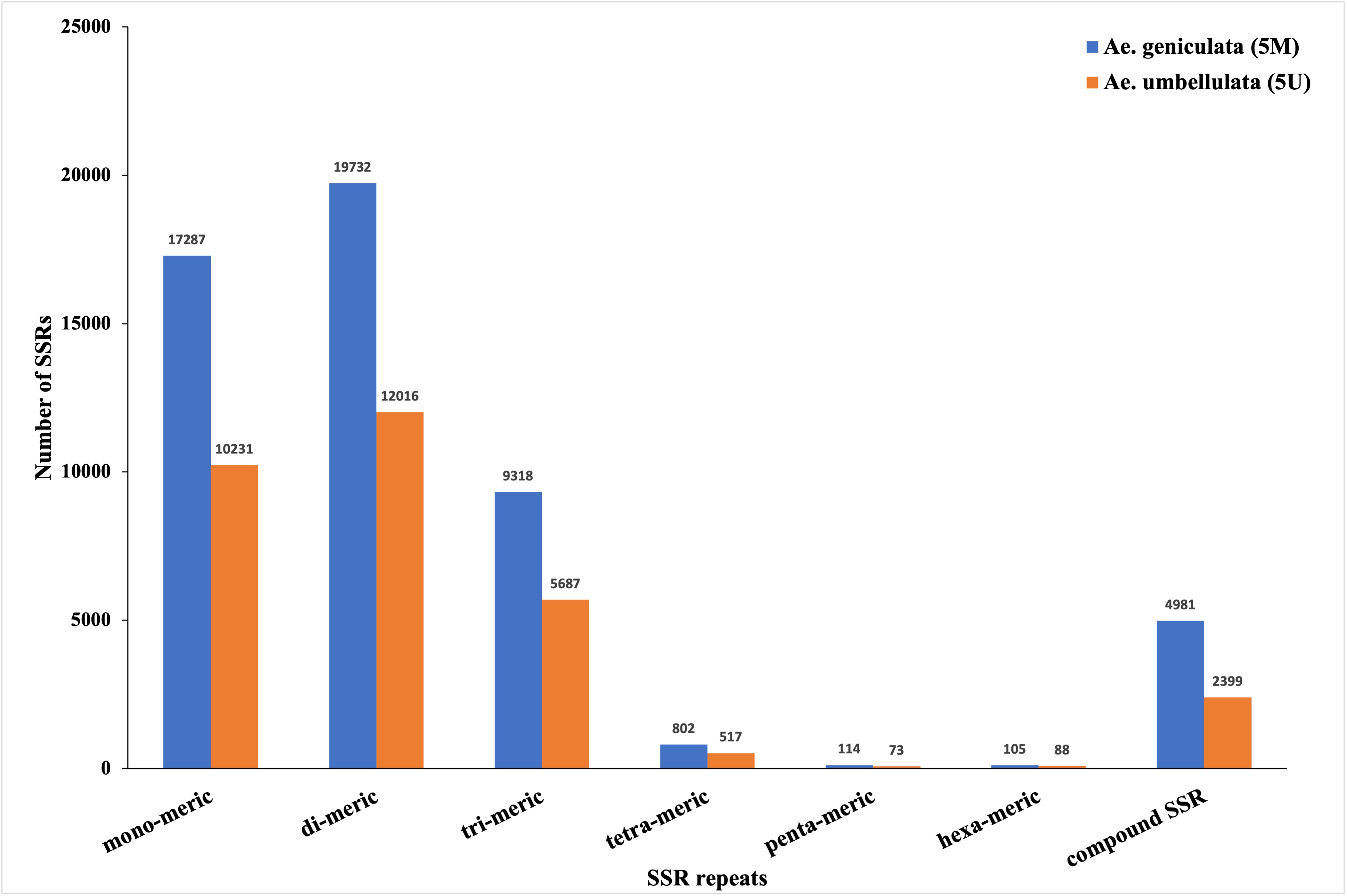

Supplement: Supplementary Figure 7 — Distribution of simple sequence repeats in Ae. geniculata (5Mg) and Ae. umbellulata (5Uu). [file Image_7.jpeg]

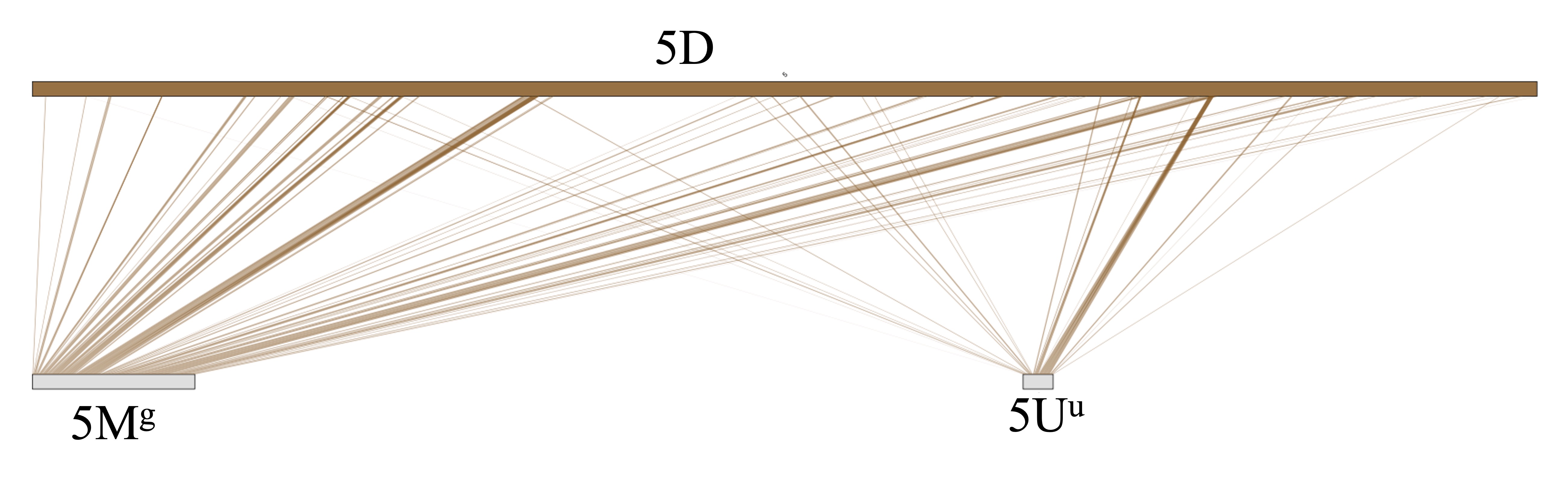

Supplement: Supplementary Figure 8 — Synteny of Ae. geniculata (5Mg) and Ae. umbellulata (5Uu) chromosomes with chromosome 5D of Chinese spring in 10Mb introgression from introgression lines. Connecting lines represents the contig location on 5D. [file Image_8.jpeg]
